# Primary healthcare providers’ perceived preparedness to respond to intimate partner violence in the public primary healthcare setting: a cross-sectional study

**DOI:** 10.1186/s12875-025-02793-2

**Published:** 2025-03-31

**Authors:** Ying Ying Chan, Noran Naqiah Hairi, Wan Yuen Choo, Zamtira Seman, Mohd Azahadi Omar, Noor Raihan Khamal, Shazimah Abdul Samad, Azah Abdul Samad, Sajaratulnisah Othman

**Affiliations:** 1 Centre for Family Health Research, Institute for Public Health, National Institutes of Health, Ministry of Health Malaysia, Shah Alam, Selangor, Malaysia; 2 Department of Social and Preventive Medicine, Faculty of Medicine, Universiti Malaya, Kuala Lumpur, Malaysia; 3 Sector for Biostatistics & Data Repository, National Institutes of Health, Ministry of Health Malaysia, Shah Alam, Selangor, Malaysia; 4 National Centre of Excellence for Mental Health (NCEMH), Ministry of Health Malaysia, Cyberjaya, Malaysia; 5 Family Health Development Division, Ministry of Health Malaysia, Putrajaya, Malaysia; 6 Department of Primary Care Medicine, Faculty of Medicine, Universiti Malaya, Kuala Lumpur, Malaysia

**Keywords:** Intimate partner violence, Primary healthcare providers, Preparedness, Readiness, Barriers, Screening, Training, PREMIS-Malay, Malaysia

## Abstract

**Background:**

Intimate partner violence (IPV) is the most prevalent form of violence against women globally, leading to various adverse health consequences. Primary healthcare providers (PHCPs) are often the first point of contact for identifying and managing IPV. However, research on PHCPs’ responses to IPV in low- and middle-income countries, including Malaysia, remains limited. This study aimed to determine the perceived preparedness to respond to IPV and its associated factors among PHCPs in Malaysia.

**Methods:**

This cross-sectional study involved 1505 PHCPs selected through multistage stratified cluster random sampling from public primary healthcare clinics in Malaysia. Data were collected via self-administered online surveys using the validated Physician Readiness to Manage Intimate Partner Violence Survey (PREMIS)-Malay tool. Complex sample analysis of descriptive data, general linear model (GLM) and logistic regression were performed. The GLM was used to determine knowledge and opinion score variables predicting the mean perceived preparedness score, whereas multivariable logistic regression identified factors associated with a good level of perceived preparedness to manage IPV. A *p*-value ≤ 0.05 was considered statistically significant.

**Results:**

Most Malaysian PHCPs (81.0%) had not received any IPV training. Only 29.1% of the PHCPs reported a good level of perceived preparedness, 12.2% had good perceived knowledge, and a mere 8.6% had good actual knowledge. The perceived and actual knowledge, workplace/self-efficacy, and staff constraints scores were positively associated with the preparedness score, whereas the victim understanding score was negatively associated with the preparedness score. Multivariable logistic regression analysis revealed that longer work experience (≥ 10 years) (AOR = 1.70, 95% CI: 1.28–2.26), prior IPV training (AOR = 1.68, 95% CI: 1.12–2.51), previous experience with IPV inquiry (AOR = 1.55, 95% CI: 1.10–2.19), good perceived knowledge (AOR = 15.21, 95% CI: 11.15–20.74), and good actual knowledge (AOR = 1.79, 95% CI: 1.10–2.94) were significantly associated with a good level of perceived preparedness.

**Conclusions:**

A high percentage of Malaysian PHCPs have not received IPV training, and only a small proportion felt they are prepared to manage IPV, with even fewer possessing adequate knowledge about IPV. These findings highlight the urgent need to prioritize IPV training programs at the primary care level to better equip PHCPs with the knowledge and skills necessary to manage IPV effectively.

**Supplementary Information:**

The online version contains supplementary material available at 10.1186/s12875-025-02793-2.

## Background

Intimate partner violence (IPV), commonly referred to as domestic violence (DV) or family violence (FV), is a major public health and social issue with far-reaching health consequences. Despite its serious health consequences, many cases of IPV remain undetected and inadequately addressed in healthcare settings. This is largely due to a lack of preparedness, training, and awareness among healthcare providers (HCPs), who may feel ill-equipped to provide adequate support and interventions [1]. Globally, more than one in three women aged 15 years or older has experienced IPV in their lifetime [2]. The World Health Organization (WHO) defines IPV as “behavior within an intimate relationship that causes physical, sexual or psychological harm, including acts of physical aggression, sexual coercion, psychological abuse and controlling behaviors” by a current or former partner [3]. Recent data from the WHO indicate that lifetime IPV prevalence estimates vary across different regions between 18% and 35%, with low-income countries reporting higher rates than high-income countries [4]. In Malaysia, the prevalence of IPV ranges widely from 4.94 to 35.9%, with psychological or emotional violence being the most common type of IPV, followed by physical and sexual violence [5]. A facility-based study reported a 12-month prevalence of 22% among women attending public primary healthcare clinics in Kuala Lumpur [6]. The National Health and Morbidity Survey (NHMS) conducted in 2022 reported a 4.4% prevalence of past-year IPV and a 7.1% prevalence of lifetime IPV among women of reproductive age [7]. However, the true prevalence of IPV is likely underreported because of the sensitive nature of the issues and their associated stigma.

Despite variations in IPV prevalence across low-, middle-, and high-income countries, the health impacts remain consistent worldwide [8, 9]. The health consequences associated with IPV are well-documented, including physical (such as fatal and nonfatal injuries) and mental health impacts (such as depression, posttraumatic stress and other anxiety disorders, suicide), as well as adverse health risk behaviors (such as sexually transmitted infections and harmful substance use) among individuals experiencing IPV [10–12]. Among women, adverse reproductive and pregnancy-related outcomes, such as unintended pregnancies, unsafe abortions, and premature births, have also been reported [10, 12]. These adverse health consequences cause women experiencing IPV to seek medical care from healthcare services more frequently than those not affected by IPV [13]. Healthcare settings such as primary care, emergency departments, and other medical subspecialties often serve as the first point of contact for IPV victims. Primary care, as the entry point to the healthcare system, places primary healthcare providers (PHCPs) in an optimal position for IPV inquiry and early detection, prevention and intervention efforts [14].

The WHO has developed clinical and policy guidelines to strengthen healthcare responses to IPV. These guidelines aim to help HCPs address the health needs of victims and guide policymakers and program managers in improving health systems by considering available resources, national policies, procedures, and support services [15]. In Malaysia, the One Stop Crisis Centre (OSCC) Policy and Guidelines [16] serve as the primary framework for managing IPV cases. Despite having a well-established OSCC system in place, the national OSCC policy focuses mainly on service provision at the secondary and tertiary hospital levels, with limited resources and engagement in the primary healthcare sector. Furthermore, no formal IPV training module has been established in Malaysia since the introduction of the OSCC [17]. There are also no clear practice standards for the identification and management of IPV at the primary healthcare level.

In primary healthcare settings, many IPV cases remain unreported or undetected due to various barriers, including the lack of preparedness, knowledge, and skills among PHCPs to handle such situations effectively [18, 19]. Inadequate readiness to manage IPV among PHCPs often results in ineffective responses to IPV disclosures and referrals, thereby preventing victims from receiving the necessary care and support. Common barriers faced by HCPs include time constraints, a lack of privacy, inadequate training, uncertainty about how to respond, and societal beliefs, which may influence their practices and limit their ability to intervene [20]. Additionally, patient-related barriers such as feelings of shame, embarrassment, fear of retaliation, and lack of trust that HCPs are capable of handling domestic abuse prevent abused women from seeking help from HCPs [21]. Without appropriate treatment and management, victims are at risk of suffering from ongoing physical, psychological, and sexual health problems.

Research in Malaysia has shown that only a small proportion of IPV victims seek help from healthcare workers, suggesting the need for greater efforts to understand and support the help-seeking process for victims [22]. Moreover, most local studies focused on IPV prevalence and associated factors [5, 6, 23, 24], with limited research addressing the identification and management of IPV [25, 26]. This study addresses this gap by providing empirical evidence on PHCPs’ preparedness and identifying key factors influencing their ability to manage IPV effectively.

The study findings can significantly bridge the gap between policy and practice by identifying real-world challenges and informing targeted intervention. Policymakers can leverage these insights to create comprehensive structured IPV training programs, strengthen primary healthcare responses, and form national IPV management policies in alignment with global best practices. Therefore, this study aims to assess PHCPs’ perceived preparedness to manage IPV in the Malaysian primary healthcare setting and to determine factors associated with their preparedness to respond to IPV cases, using the validated Malay version of the Physician Readiness to Manage Intimate Partner Violence Survey (PREMIS-Malay) tool [27].

## Methods

### Study design and study population

This was a cross-sectional study in which a multistage stratified cluster sampling design was used to select a nationally representative sample of primary healthcare providers (PHCPs) from five states in Malaysia. Malaysia comprises 13 states and 3 federal territories, which can be divided into five regions: Northern, East Coast, Central, Southern, and East Malaysia. To maximize efficiency, five states were randomly chosen to represent the five different regions in Malaysia: Kedah (Northern), Kelantan (East Coast), Selangor (Central), Malacca (Southern), and Sarawak (East Malaysia). The sampling frame containing a list of public primary care clinics was obtained from the Family Health Development Division, Ministry of Health Malaysia. For each selected state, all public health clinics, including maternal and child health clinics, were stratified by urban-rural classification, followed by random selection of urban and rural districts via a simple random sampling technique. Both urban and rural health clinics within the selected districts were then randomly chosen via a systematic probability-proportional-to-size (PPS) sampling technique. The eligibility criteria included PHCPs who serve as the first point of contact for patients in primary healthcare clinics, such as Family Medicine Specialists (FMS), Medical Officers (MO), Assistant Medical Officers (AMO), Staff Nurses (SN) and Community Nurses (CN), and who had worked in public primary care facilities for a minimum period of at least 6 months at the time of the survey. In this study, a total of 60 health clinics (22 urban clinics and 38 rural clinics) were randomly selected across the five states. All eligible PHCPs from the selected health clinics were invited to participate in the survey.

### Sample size determination

The sample size was calculated via the single population proportion sample size calculation formula. In reference to related findings from a previous local study [25], the maximum proportion of PHCPs who perceived prepared to respond to IPV is assumed to be 50%, with a Z value of 1.96, a design effect of 2, a marginal error of 5%, and a 50% nonresponse rate; the calculated sample size was 1536. Additionally, G*Power was used to estimate the sample size for a multiple linear regression with an alpha of 0.05, a power of 0.80, an anticipated effect size of 0.02, 16 predictors, and a 50% nonresponse rate; the required sample size was 1954. Thus, the larger estimate was selected, and the final sample size needed for this study was determined to be 2000.

### Survey response rate

In this study, overall response rate was defined as the proportion of PHCPs who achieved a “complete response” (100% of applicable questions answered) or “usable response” (more than half but < 100% of all applicable questions answered, with all key items under Section A to C of the survey questionnaire answered) among the eligible PHCPs. A “usable response” indicates that the respondent has provided sufficient data for inclusion in the analysis, despite not answering every question on the survey. A total of 1505 out of 2326 eligible PHCPs from five states in Malaysia provided a complete or usable response, resulting in an overall response rate of 64.7%. This response rate is considered satisfactory for an online survey. A non-response bias analysis was performed to determine whether non-responders differed systematically from responders. No significant differences were found in key demographic and professional characteristics, indicating that non-response bias was not a concern in this study.

### Study instrument and data collection

The instrument used was the Malay version of the Physician Readiness to Manage Intimate Partner Violence Survey (PREMIS-Malay) tool, which was previously adapted and validated for use in the Malaysian primary healthcare setting [27]. This tool has demonstrated promising psychometric properties, with acceptable to good levels of internal consistency (Cronbach’s alpha: 0.59–0.98) and satisfactory to high levels of test-retest reliability (intraclass correlation coefficients; ICC = 0.52–0.85) across all subscales [27]. The PREMIS-Malay tool is a translated and modified version of the original English version PREMIS questionnaire developed by Short et al. (2006) [28]. The PREMIS-Malay questionnaire comprises six sections: (A) Respondent profile, (B) Background (including perceived preparedness and knowledge), (C) Actual knowledge, (D) Opinions, (E) Practice issues, and (F) Personal experience. Both the English and Malay versions of the PREMIS questionnaire are provided in Supplementary Files 1 and 2, respectively. For this study, the self-administered PREMIS-Malay questionnaire was developed into an online survey via the Research Electronic Data Capture (REDCap) platform [29, 30]. Only the Malay version was distributed in the online survey among PHCPS to ensure relevance for the local context and setting. The questionnaire took approximately 20–25 min to complete.

Data collection was carried out between June and August 2023. The online survey data were collected and managed via the secure, web-based REDCap application. A liaison officer was appointed at each selected health clinic to coordinate data collection and distribute a digital flyer containing the survey link and QR code to healthcare staff at their respective clinics. Eligible staff members responded to the online survey via mobile devices, computers, or laptops with internet access. The respondents had the option to save their responses and complete the survey later. To increase response rates, automated email reminders were sent daily up to three times for incomplete responses. All eligible PHCPs provided online informed consent prior to the start of the survey. A pretest was conducted prior to actual data collection.

## Measures

### Dependent variable

The “Perceived preparedness” subscale measures how well respondents believe that they are prepared to manage IPV through nine items, each rated on a 7-point Likert scale ranging from “1 = not prepared” to “7 = quite well prepared”. The overall perceived preparedness score was determined by calculating the mean score across all items in the subscale. To facilitate interpretation, the mean percentage score was categorized into three levels based on cutoffs from a previous study: poor (< 50%), fair (50 to < 75%), and good (≥ 75%) [31]. For further analysis purposes, a binary outcome variable was created by grouping the levels of perceived preparedness into “good” versus “poor/fair”, which enhances interpretability and supports intervention planning. Continuous analysis captured how different factors affect the overall mean score, while categorical analysis identified specific groups that might require targeted interventions. By presenting “Perceived preparedness” as both a continuous mean score and a categorical variable, this approach provides a more comprehensive understanding of the outcome variable.

### Independent variables

Data were collected on the following independent variables: sociodemographic variables, personal and work-related variables, perceived and actual knowledge, and opinion subscale variables.

The sociodemographic variables included clinic locality (urban/rural), age, sex, ethnicity, marital status (married, never married/separated/divorced/widowed), job position (FMS, MO, AMO, SN and CN), and educational level (certified, diploma, bachelor, master). Age was categorized into two groups: <40 years and ≥ 40 years. Ethnicity was classified into Malays, Chinese, Indians, and ‘Others’ (including Bumiputeras from Sabah and Sarawak, and other minority ethnic groups).

The personal and work-related variables included the number of years in primary care practice (< 10/≥10 years), average number of patients cared for per week (< 50/≥50), prior IPV training (yes/no), previous experience with IPV inquiry (yes/no), identification/disclosure of IPV in the last six months (yes/no), history of personal experience with IPV (yes/no), and history of being a witness of IPV directed toward a family member (yes/no). Two questions related to personal and family IPV experience were adapted from a modified healthcare student version of the PREMIS [32]: (a) “Have you ever experienced physical violence, sexual abuse, emotional abuse, intimidation, economic deprivation, or threats of violence in an intimate partner relationship?” and (b) “Have you ever witnessed physical violence, sexual abuse, or psychological abuse directed toward a family member?”

Perceived knowledge was measured through 14 items that ask respondents to indicate how much they feel they know about IPV via a 7-point Likert scale (“1 = Nothing” to “7 = Very much”). Actual knowledge about IPV was measured through a combination of six multiple-choice questions and 11 ‘True/False’ statements. Each knowledge question was recoded to a dichotomous variable (0 = incorrect, 1 = correct). A summative knowledge score was computed based on the number of correct responses. The “Actual knowledge” items covered questions about the strongest risk factor for becoming a victim of IPV, batterers, warning signs, reasons for an IPV victim not leaving a violent relationship, the most appropriate ways to ask about IPV, and general concepts about IPV. The mean scores and mean percentage scores for both perceived and actual knowledge were calculated, and the mean percentage scores were further grouped into poor (< 50%), fair (50 to < 75%), and good (≥ 75%) levels.

The PREMIS-Malay “Opinions” scale consists of 25 items graded on a 7-point Likert scale from “1 = Strongly disagree” to “7 = Strongly agree”, of which seven subscales were formed to measure the attitudes and beliefs of respondents regarding workplace/self-efficacy (6 items), staff preparation (4 items), legal requirements (3 items), staff constraints (4 items), alcohol/drugs (3 items), IPV enquiry/screening (3 items), and victim understanding (2 items). Both mean scores and mean percentage scores were computed for each of the opinion subscales. Detailed information on the individual items contributing to the seven opinion subscales or factors is reported in our previously published work [27].

### Data analysis

The collected data were analyzed using IBM SPSS version 28. Prior to any analysis, the data were checked for outliers, missing values, and distribution patterns. All variables used in the analysis had missing data of less than 5%, which was considered inconsequential. Little’s MCAR test result was not significant, indicating that the missing data were completely at random. Therefore, no imputation was performed, and missing data was handled by listwise deletion or available case analysis.

Descriptive statistics were used to examine the frequency distribution of participants’ sociodemographic, personal and work-related characteristics. The mean scores and mean percentage scores for the PREMIS-Malay subscales were computed based on the scoring system provided in the original PREMIS toolkit (available online at www.ajpm-online.net) and in accordance with our previously published work on the validation of the PREMIS-Malay tool [27]. For further analysis via inferential statistics, categories with small cell sizes, such as marital status, job positions (grouped into provider type), and educational level, were collapsed with related categories.

Complex Samples General Linear Model (CSGLM) regression analysis was performed, with knowledge and opinion subscales as predictors, and the perceived preparedness score as the continuous outcome variable. Sociodemographic, personal and work-related variables were treated as covariates. The strength of associations between the knowledge and opinion scores with the perceived preparedness score was quantified using regression coefficients (*β*) with 95% confidence intervals (CI), and the *p*-values for each factor in the final model were reported. Additionally, factors associated with a good level of perceived preparedness (binary outcome variable) to response to IPV were studied using univariable and multivariable logistic regression analyses. Chi-square tests were used to examine the relationships between the studied variables and perceived preparedness (good vs. poor/fair). Variables with a *p-*value of < 0.25 in the univariable analysis were included in the multivariable logistic regression model [33]. All assumptions for linear and logistic regression analyses such as goodness of fit, multicollinearity and interaction effects were checked. Two-way interactions (e.g., between IPV knowledge and prior IPV training) were tested in the model. However, none were statistically significant (*p* > 0.05), indicating no significant interaction effect. The final multivariable model was adjusted for potential confounding factors such as clinic locality, age, ethnicity, and educational level, with statistical significance set at *p* < 0.05. The results were presented as crude and adjusted odds ratios (ORs) with 95% CI. All the statistical analyses were conducted using a complex sampling design, accounting for both sample weights and study design considerations.

### Ethical consideration

This study was registered with the National Medical Research Registry (NMRR) and approved by the Medical Research and Ethics Committee (MREC), Ministry of Health Malaysia, with the registration number NMRR ID-22-01067-HDU. Online informed consent was obtained from all participants prior to the start of the survey. All responses were anonymized to maintain privacy and data protection. All data were securely stored in a password-protected system, accessible only to the research team, in compliance with applicable laws and/or regulations.

## Results

### Descriptive analyses

Table 1 presents the sociodemographic, personal and work-related characteristics of the participants. Approximately 60% of the participants were from urban clinics. The majority of the participants were below 40 years old (62.6%), with a mean age of 38.1 years (SD = 7.0; age range, 20–60 years). Most of the participants were females (81.4%), Malays (77.1%), and were married (85.6%). With regards to job position, 30.5% were Staff Nurse (SN), 26.8% were Medical Officer (MO), 24.7% were Community Nurse (CN), 16.0% were Assistant Medical Officer (AMO), and the remaining 2.1% were Family Medicine Specialist (FMS). In terms of education level, nearly half of the participants had a diploma (45.3%), followed by a bachelor’s degree (29.5%), a certificate (22.7%), and a master’s degree (2.5%). The percentage of participants with less than 10 years in primary care practice (49.0%) was almost equal to that of those with more than 10 years of experience in primary care (51.0%). The length of working experience ranged between 1 and 34 years (mean = 9 years). Most of the participants did not receive any training on IPV (81.0%). Nearly three-fourths of the participants (74.1%) reported taking care of an average of 50 or more patients per week. Over half of the participants (66.2%) had never asked patients about IPV. Only 16.0% of the participants reported having encountered identification/disclosure of IPV in the last six months, whereas 84.0% reported none. A small percentage of participants (5.6%) reported having personally experienced IPV, whereas approximately 12% had witnessed physical, sexual or psychological abuse directed toward a family member (Table 1).

**Table 1:** Sociodemographic, personal and work-related characteristics of the participants (*n* = 1505)

Characteristics	Unweighted count (*n*)	Estimated population (*N*)	Percentage % (95% CI)
Clinic locality			
Urban	834	17,708	58.7 (46.5–69.8)
Rural	671	12,485	41.3 (30.2–53.5)
Age group (years)			
<40	929	18,911	62.6 (58.6–66.5)
≥40	576	11,281	37.4 (33.5–41.4)
Sex			
Male	283	5610	18.6 (16.5–20.8)
Female	1222	24,583	81.4 (79.2–83.5)
Ethnicity			
Malay	1172	23,272	77.1 (66.6–85.0)
Chinese	76	1386	4.6 (3.0–7.0)
Indian	67	1164	3.9 (2.7–5.4)
Others^a^	190	4370	14.5 (7.4–26.3)
Marital status			
Married	1278	25,850	85.6 (83.0–87.9)
Never married/separated/divorced/widowed	227	4343	14.4 (12.1–17.0)
Job position			
Family Medicine Specialist (FMS)	35	624	2.1 (1.5–2.9)
Medical Officer (MO)	451	8090	26.8 (24.0–29.8)
Assistant Medical Officer (AMO)	225	4819	16.0 (13.2–19.1)
Staff Nurse (SN)	432	9200	30.5 (27.7–33.4)
Community Nurse (CN)	362	7460	24.7 (21.7–28.0)
Highest education level			
Certified	332	6852	22.7 (19.9–25.7)
Diploma	644	13,687	45.3 (42.0–48.7)
Bachelor	487	8914	29.5 (26.6–32.6)
Master	42	740	2.5 (1.6–3.7)
Number of years in primary care practice			
<10	742	14,785	49.0 (44.8–53.1)
≥10	763	15,409	51.0 (46.9–55.2)
Average number of patients cared for per week			
<50	398	7832	25.9 (21.8–30.6)
≥50	1107	22,361	74.1 (69.4–78.2)
Ever received IPV training			
Yes	281	5727	19.0 (17.2–20.8)
No	1224	24,467	81.0 (79.2–82.8)
Ever asked patients about IPV			
Yes	523	10,050	33.8 (30.4–37.5)
No	960	19,645	66.2 (62.5–69.6)
Identification/disclosure of IPV in the last 6 months			
Yes	242	4756	16.0 (13.9–18.4)
No	1241	24,938	84.0 (81.6–86.1)
Ever experienced with IPV^b^			
Yes	78	1615	5.6 (4.4–7.1)
No	1374	27,418	94.4 (92.9–95.6)
Ever witnessed physical, sexual or psychological abuse directed towards a family member			
Yes	171	3395	11.7 (9.9–13.7)
No	1281	25,638	88.3 (86.3–90.1)

***Notes***: Some variables have missing values (less than 5%). Percentages are based on total number of responses and weighted by sampling weights. 95% CI = 95% confidence interval

^a^ ‘Others’ ethnic group includes Bumiputeras from Sabah and Sarawak, and other minority ethnic groups

^b^ Ever experienced physical violence, sexual abuse, emotional abuse, intimidation, economic deprivation or threats of violence in an intimate partner relationship

The mean scores and mean percentage scores for the PREMIS-Malay scales/subscales are presented in Table 2. The “theoretical” score refers to the possible minimum and maximum raw scores that participants could achieve by computing specific items related to each subscale, whereas the “observed” score represents the actual total minimum and maximum scores obtained from participants’ real responses. Presenting both the “theoretical” and “observed” scores allows a clearer understanding of how participants performed relative to the full scoring range of each PREMIS subscale. The mean perceived preparedness score was 4.38 ± 1.42, with a mean percentage score of 62.53 ± 20.23. The mean score for perceived knowledge was 3.82 ± 1.29, with a mean percentage score of 54.57 ± 18.37. Additional information on the distribution and mean score for each item of the “Perceived preparedness” and “Perceived knowledge” scales is provided in Supplementary File 3 (Tables S1 and S2). For actual knowledge, the mean score was 16.19 ± 5.43, with a mean percentage score of 50.58 ± 16.97. For the opinion subscales, the mean scores ranged from 3.36 to 5.68, corresponding to mean percentage scores between 48.00 and 76.78. Additionally, the mean score for practice issues was 11.50 ± 6.27, with a relatively low mean percentage score of 19.17 ± 10.45 (Table 2).

**Table 2:** Mean scores and mean percentage scores for the PREMIS-Malay scales/subscales

Scales and subscales	Theoretical Minimum / Maximum	Observed Minimum / Maximum	Mean score ± SD	Mean percentage score ± SD
**Background**				
Perceived preparedness	9–63	9–63	4.38 ± 1.42	62.53 ± 20.23
Perceived knowledge	14–98	14–98	3.82 ± 1.29	54.57 ± 18.37
**Actual knowledge**				
Actual knowledge	0–38	2–32	16.19 ± 5.43	50.58 ± 16.97
**Opinions**				
Workplace/Self-efficacy	6–42	6–42	4.07 ± 1.04	58.18 ± 14.88
Staff preparation	4–28	4–28	3.40 ± 1.22	48.52 ± 17.35
Legal requirements	3–21	3–21	5.68 ± 1.17	76.78 ± 16.76
Staff constraints	4–28	4–28	4.20 ± 1.02	60.05 ± 14.60
Alcohol/drugs	3–21	5–21	4.36 ± 0.56	62.28 ± 8.03
IPV enquiry/screening	3–21	5–21	4.07 ± 0.67	58.14 ± 9.53
Victim understanding	2–14	2–14	3.36 ± 1.15	48.00 ± 16.40
**Practice issues**				
Practice issues	0–60	1–36	11.50 ± 6.27	19.17 ± 10.45

***Notes***: N/A = not applicable; SD = standard deviation

**Fig. 1:**
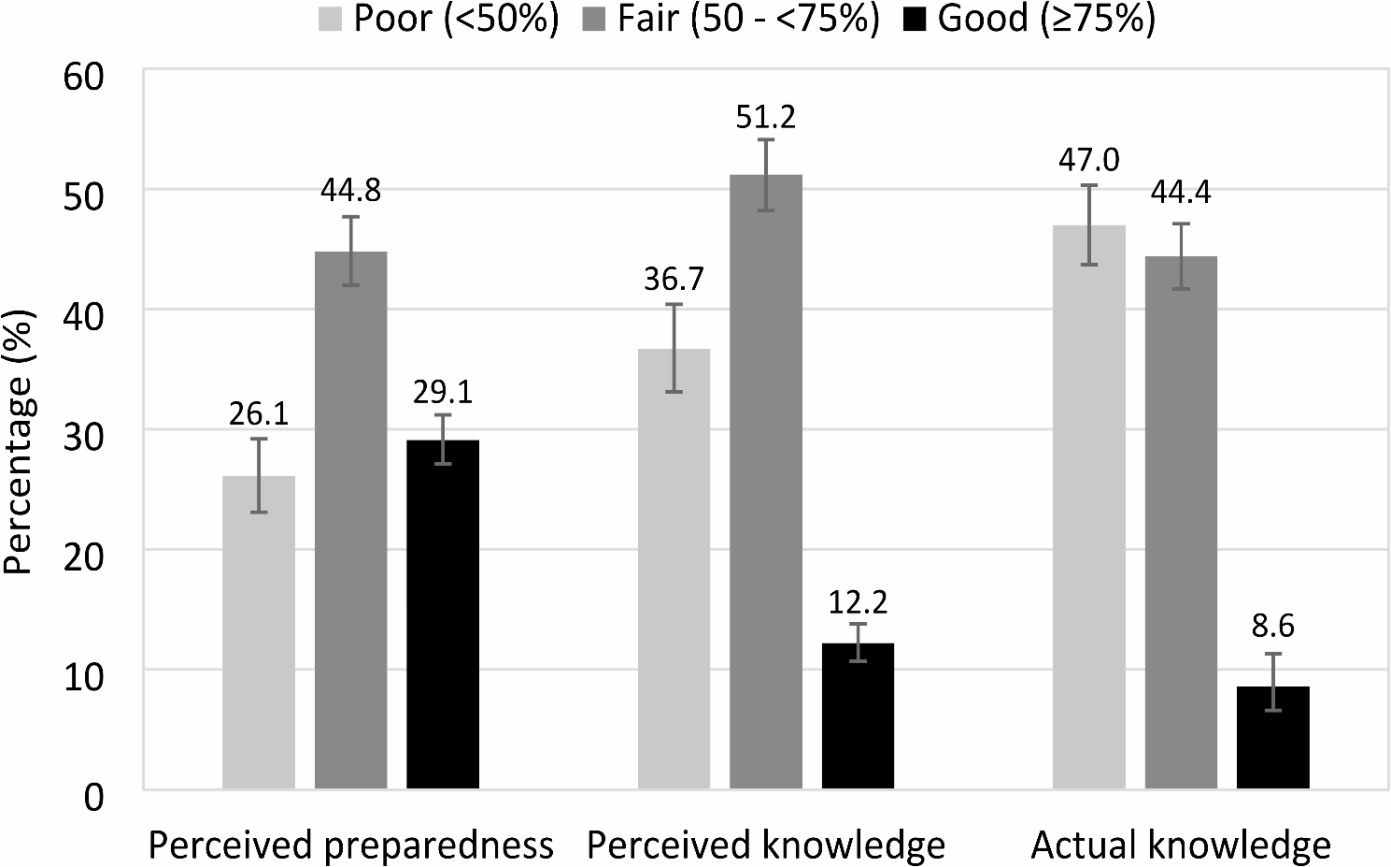
Participants’ levels of perceived preparedness, perceived knowledge, and actual knowledge

As shown in Fig. 1, around three in ten participants (29.1%, 95% CI: 27.1–31.2) had a good level of perceived preparedness, and only about one in ten participants reported having a good level of perceived knowledge (12.2%, 95% CI: 10.7–13.8) and actual knowledge (8.6%, 95% CI: 6.6–11.3). Approximately half of the participants had a fair level of perceived preparedness (44.8%, 95% CI: 42.0-47.7) and perceived knowledge (51.2%, 95% CI: 48.2–54.1), but a poor level of actual knowledge (47.0%, 95% CI: 43.7–50.3).

### Inferential analyses

To determine the associations between knowledge and opinion scores with perceived preparedness score, while controlling for all other variables in the model, the results of Complex Samples General Linear Model (CSGLM) regression analysis are presented in Table 3. Specifically, perceived knowledge (*β* = 0.65, *p* < 0.001), actual knowledge (*β* = 0.01, *p* = 0.009), workplace/self-efficacy (*β* = 0.20, *p* < 0.001), and staff constraints score (*β* = 0.08, *p* = 0.004) were significantly positively associated with the perceived preparedness score. A significant and negative association was observed between the victim understanding score and the perceived preparedness score (*β* = -0.10, *p* = 0.029) (Table 3).

**Table 3:** Complex samples general linear model (CSGLM) regression analysis of knowledge and opinions score variables predicting the mean perceived preparedness score (*n* = 1505)

Independent variables	Regression coefficient (95% CI)^a^	S.E	*p*-value
Intercept	-0.50 (-1.35, 0.35)	0.42	0.243
Perceived knowledge score	0.65 (0.56, 0.73)	0.04	**< 0.001**
Actual knowledge score	0.01 (0.00, 0.02)	0.01	**0.009**
Workplace/Self-efficacy score	0.20 (0.11, 0.29)	0.05	**< 0.001**
Staff preparation score	-0.02 (-0.07, 0.04)	0.03	0.563
Legal requirements score	0.06 (-0.03, 0.15)	0.04	0.182
Staff constraints score	0.08 (0.03, 0.14)	0.03	**0.004**
Alcohol/drugs score	0.06 (-0.03, 0.15)	0.05	0.211
IPV enquiry/screening score	0.10 (-0.01, 0.22)	0.06	0.076
Victim understanding score	-0.10 (-0.19, -0.01)	0.04	**0.029**

***Notes***: ^a^ Model adjusted for socio-demographic, personal and work-related variables. 95% CI = 95% confidence interval; S.E = Standard Error

Goodness of fit: R-squared = 0.544; Adjusted R-squared = 0.541

Table 4 shows the associations between sociodemographic, personal and work-related characteristics, perceived and actual knowledge with perceived preparedness to manage IPV (good vs. poor/fair) using the chi-square test. The number of years in primary care practice (*p* = 0.002), average number of patients cared for per week (*p* = 0.001), prior IPV training (*p* < 0.001), previous experience with IPV inquiry (*p* < 0.001), identification/disclosure of IPV in the last 6 months (*p* < 0.001), and personal experience with IPV (*p* = 0.032) were significantly associated with a good level of perceived preparedness. Both good perceived knowledge and actual knowledge were significantly associated with good perceived preparedness at a *p*-value of < 0.001.

**Table 4:** Associations between sociodemographic, personal and work-related characteristics, perceived and actual knowledge with perceived preparedness to manage IPV (good vs. poor/fair) (*n* = 1505)

Characteristics	Perceived preparedness to manage IPV			*p*-value^a^
	Good (*n* = 440)		Poor/Fair (*n* = 1065)	
	*n* (%)		*n* (%)	
Clinic locality				0.198
Urban	255 (30.3)		579 (69.7)	
Rural	185 (27.4)		486 (72.6)	
Age group (years)				0.150
<40	264 (27.9)		665 (72.1)	
≥40	176 (31.1)		400 (68.9)	
Sex				0.685
Male	84 (30.0)		199 (70.0)	
Female	356 (28.9)		866 (71.1)	
Ethnicity				0.252
Malay	347 (29.8)		825 (70.2)	
Chinese	19 (23.2)		57 (76.8)	
Indian	27 (36.7)		40 (63.3)	
Others	47 (25.5)		143 (74.5)	
Marital status				0.701
Married	378 (29.3)		900 (70.7)	
Never married/separated/ divorced/widowed	62 (28.0)		165 (72.0)	
Provider type				0.616
Doctors (FMS & MO)	140 (28.2)		346 (71.8)	
Non-doctors (AMO, SN & CN)	300 (29.5)		719 (70.5)	
Highest education level				0.062
Certified	112 (33.7)		220 (66.3)	
Diploma	173 (26.7)		471 (73.3)	
Bachelor/ Master	155 (29.2)		374 (70.8)	
Number of years in primary care practice				**0.002**
<10	190 (25.2)		552 (74.8)	
≥10	250 (32.9)		513 (67.1)	
Average number of patients cared for per week				**0.001**
<50	135 (34.0)		263 (66.0)	
≥50	305 (27.4)		802 (72.6)	
Ever received IPV training				**< 0.001**
Yes	124 (45.4)		157 (54.6)	
No	316 (25.3)		908 (74.7)	
Ever asked patients about IPV				**< 0.001**
Yes	204 (39.1)		319 (60.9)	
No	228 (23.8)		732 (76.2)	
Identification/disclosure of IPV in the last 6 months				**< 0.001**
Yes	92 (36.7)		150 (63.3)	
No	340 (27.5)		901 (72.5)	
Ever experienced with IPV				**0.032**
Yes	26 (39.1)		52 (60.9)	
No	397 (28.5)		977 (71.5)	
Ever witnessed physical, sexual or psychological abuse directed towards a family member				0.156
Yes	54 (35.4)		117 (64.6)	
No	369 (28.2)		912 (71.8)	
Perceived knowledge				**< 0.001**
Poor/Fair	285 (21.6)		1040 (78.4)	
Good	155 (83.3)		25 (16.7)	
Actual knowledge				**< 0.001**
Poor/Fair	382 (27.6)		992 (72.4)	
Good	58 (45.1)		73 (54.9)	

***Notes***: ^a^ Chi-square test of independence was conducted

The results of both the univariable and multivariable logistic regression analyses are presented in Table 5. In the final multivariable model, after adjusting for potential confounding variables such as clinic locality, age, ethnicity, and educational level, the results revealed that participants who had working experience of 10 years or more in primary care (AOR = 1.70, 95% CI: 1.28–2.26), had ever received IPV training (AOR = 1.68, 95% CI: 1.12–2.51), and had ever asked patients about IPV (AOR = 1.55, 95% CI: 1.10–2.19) were approximately twice more likely to have a good level of perceived preparedness. Participants with a good level of perceived knowledge were 15 times more likely to have a good level of perceived preparedness (AOR = 15.21, 95% CI: 11.15–20.74), whereas participants with a good level of actual knowledge were approximately twice more likely to have good perceived preparedness (AOR = 1.79, 95% CI: 1.10–2.94).

**Table 5:** Univariable and multivariable logistic regression analysis for factors associated with a good level of perceived preparedness to respond to IPV among PHCPs (*n* = 1505)

**Independent variables**	Univariable analysis			Multivariable analysis^a^		
	COR	95% CI	*p*-value	AOR	95% CI	*p*-value
Clinic locality						
Urban	1			1		
Rural	0.87	(0.70, 1.08)	0.198	0.81	(0.61, 1.06)	0.125
Age group (years)						
<40	1			1		
≥40	1.17	(0.94, 1.44)	0.15	0.98	(0.68, 1.40)	0.902
Sex						
Male	1					
Female	0.95	(0.74, 1.23)	0.685		–	
Ethnicity						
Malay	1			1		
Chinese	0.71	(0.40, 1.27)	0.246	0.68	(0.35, 1.34)	0.262
Indian	1.37	(0.69, 2.70)	0.358	1.1	(0.47, 2.62)	0.82
Others	0.81	(0.60, 1.09)	0.153	0.86	(0.57, 1.30)	0.465
Marital status						
Married	1					
Never married/ separated/ divorced/widowed	0.94	(0.67, 1.31)	0.701		–	
Provider type						
Doctors (FMS & MO)	0.94	(0.74, 1.20)	0.616		–	
Non-doctors (AMO, SN & CN)	1					
Highest education level						
Certified	1			1		
Diploma	0.72	(0.54, 0.96)	**0.027**	0.79	(0.56, 1.10)	0.153
Bachelor/ Master	0.81	(0.63, 1.04)	0.098	0.81	(0.60, 1.11)	0.185
Number of years in primary care practice						
<10	1			1		
≥10	1.46	(1.16, 1.83)	**0.002**	1.7	(1.28, 2.26)	**< 0.001**
Average number of patients cared for per week						
<50	1			1		
≥50	0.73	(0.61, 0.88)	**0.001**	0.82	(0.65, 1.04)	0.097
Ever received IPV training						
Yes	2.46	(1.79, 3.38)	**< 0.001**	1.68	(1.12, 2.51)	**0.014**
No	1			1		
Ever asked patients about IPV						
Yes	2.05	(1.65, 2.56)	**< 0.001**	1.55	(1.10, 2.19)	**0.013**
No	1			1		
Identification/disclosure of IPV in the last						
6 months						
Yes	1.53	(1.22, 1.92)	**< 0.001**	0.96	(0.66, 1.40)	0.829
No	1			1		
Ever experienced with IPV						
Yes	1.62	(1.04, 2.51)	**0.034**	1.1	(0.56, 2.13)	0.783
No	1			1		
Ever witnessed physical, sexual or psychological abuse directed towards a family member						
Yes	1.4	(0.88, 2.23)	0.157	1.1	(0.60, 1.90)	0.822
No	1			1		
Perceived knowledge						
Poor/Fair	1			1		
Good	18.07	(13.17, 24.78)	**< 0.001**	15.21	(11.15, 20.74)	**< 0.001**
Actual knowledge						
Poor/Fair	1			1		
Good	2.15	(1.51, 3.08)	**< 0.001**	1.79	(1.10, 2.94)	**0.021**

***Notes***: Independent variables with *p*-value < 0.25 or important were included in the multivariable logistic regression. Multicollinearity and interactions were checked and not found. COR = crude odds ratio; AOR = adjusted odds ratio; 95% CI = 95% confidence interval

^a^ Classification table = 78.8%. Cox and Snell R Square = 0.189; Nagelkerke R Square = 0.270; McFadden R Square = 0.174

## Discussion

This study aimed to identify factors associated with perceived preparedness to respond to IPV among PHCPs in the Malaysian primary healthcare setting. The study also examined PHCPs’ current levels of perceived preparedness, perceived knowledge and actual knowledge about IPV, using the validated PREMIS-Malay tool.

Overall, a large proportion of participants in this study (81.0%) had not received training and education about IPV. This finding was consistent with previous studies conducted in various settings [18, 31, 34–39], indicating that the lack of IPV training experience among HCPs is a common issue. The findings also revealed that approximately three out of ten (29.1%) Malaysian PHCPs had a good level of perceived preparedness, whereas only about one in ten demonstrated good levels of perceived and actual knowledge (12.2% and 8.6%, respectively). Similar findings have been reported in Saudi Arabia and elsewhere [1, 31, 40, 41]. Compared with other previous studies that used the PREMIS questionnaire, there are some variations in the reported scores for perceived preparedness, perceived and actual knowledge, opinions, and practice issues among different healthcare population samples, including perinatal care providers [42], pharmacists [43] and community health workers [44]. The variations in findings across different studies could be attributed to different study populations and settings. Moreover, researchers in previous studies have adapted and modified the PREMIS questionnaire to suit the context of their research, which potentially influences the study findings.

In this study, most participants exhibited poor or fair levels of perceived and actual knowledge, which may influence their preparedness and practices in managing IPV. The findings also revealed that only a small percentage of participants had experience in asking patients about IPV (33.8%) or had encountered identification/disclosure of IPV in the last six months (16.0%). These findings could be explained by the fact that routine enquiry or screening for IPV is not part of standard practice in primary healthcare in Malaysia. The latest WHO guidelines advise against universal screening or routine enquiry for IPV, as it has not led to better outcomes for women. Instead, they recommend that clinicians screen women who are at high risk or exhibit signs or symptoms indicative of possible IPV [15, 45]. Additionally, IPV victims often seek help from informal support systems, such as family members or friends, rather than through formal services [46]. Many cases of IPV are unreported and remain undetected by HCPs. Therefore, there is a pressing need to increase knowledge and awareness of IPV care and management among both HCPs and patients.

The present study identified both perceived and actual knowledge as significant predictors of perceived preparedness to manage IPV, indicating that knowledge is a crucial prerequisite for feeling more prepared in IPV management. In terms of the participants’ opinions, higher scores in workplace/self-efficacy and staff constraints were significantly associated with a higher perceived preparedness score. These findings suggest that PHCPs who acknowledge the importance of a supportive work environment and being confidence in addressing IPV feel more prepared in managing IPV [47]. Facing constraints or barriers in clinical settings can be challenging for healthcare staff. However, this may serve as an opportunity for PHCPs to develop valuable skills and gain experience in overcoming staff constraints, which could inadvertently contribute to their preparedness to manage IPV cases.

Conversely, a negative association was found between the victim understanding score and perceived preparedness score, indicating that PHCPs with a limited understanding of victims’ experiences and needs (lower victim understanding) felt more prepared in managing IPV (higher perceived preparedness), or vice versa. This finding reflects that PHCPs with a better understanding of victims’ experiences may be more aware of the complexities of IPV and its challenges, leading to a lower perceived preparedness score. Other possible explanations include cognitive biases (e.g., biased thinking patterns, poor decision-making, or irrational judgments) [48] and the emotional burden of handling IPV cases (e.g., stress, burnout, frustration) [49], which may influence PHCPs’ perceptions on IPV victims, thereby affecting their preparedness to respond effectively. A similar result was reported in a previous study in which the PREMIS tool was used among community health workers [44]. However, comparisons of findings for “Opinions” subscales or factors across different studies using the PREMIS tool should be interpreted with caution because there may be variations in factor structure and the specific items included within each factor [27, 50].

In addition, this study identified several important factors significantly associated with a good level of perceived preparedness to manage IPV among Malaysian PHCPs. PHCPs with longer work experience in primary care (10 years or more), prior IPV training, and previous experience with IPV inquiry were significantly more likely to report a good level of perceived preparedness to respond to IPV. These findings can be explained by the ‘novice to expert theory’, which describes how clinical experience and skill acquisition over time impact nurses’ knowledge and expertise [51]. By applying the Benner’s novice to expert theory, it is believed that Malaysian PHCPs could develop skills and gain understanding of IPV care provision through years of work experience in primary care practices, ultimately leading to a state of better preparedness in addressing IPV. Similarly, a study conducted in Tanzania reported that providers with longer work experience have a higher likelihood of receiving IPV-related training [18]. Another study by Gutmanis et al. (2007) revealed that both IPV training and professional experience (i.e., frequency of abuse disclosure in the past one year) are associated with increased feelings of preparedness and self-confidence [52]. Previous studies have also consistently highlighted the relationship between IPV training and perceived preparedness, indicating that previous IPV education and training can improve providers’ knowledge, attitudes and preparedness to manage IPV [18, 31, 34, 36, 38].

In the current study, personal experience with IPV was associated with 1.1 higher odds of feeling more prepared in addressing IPV compared to those without any personal history of IPV experience, after controlling for sociodemographic and other independent variables. However, this association was not statistically significant. A similar finding was observed in a study conducted among nursing students, where students with a history of any IPV exposure were 1.6 times more likely to feel well-prepared to manage IPV compared to those with no history of IPV exposure, though the association also lacked statistical significance [53]. Another study by McLindon et al. (2019) suggested that health professionals’ personal experiences of IPV could facilitate them to provide better clinical care for patients experiencing violence compared to those without such experience [54]. However, personal IPV experience might also influence professional behavior, potentially leading some HCPs to avoid discussing this sensitive topic with patients [55]. The extent to which personal experience affects HCPs’ approach to managing IPV remains unclear. Further research is needed to qualitatively explore how personal experiences with IPV could affect providers’ preparedness to manage IPV.

The present study also found that PHCPs with good levels of perceived knowledge and actual knowledge were significantly more likely to report a good level of perceived preparedness, after controlling for sociodemographic and other potential confounding variables in the final model. This finding suggests that the more knowledgeable PHCPs are regarding the identification and management of IPV, the more prepared they perceived themselves to be. Improved knowledge and training about IPV have been shown to translate into changes in attitudes and practices among HCPs. A recent study conducted in the United States indicated that the number of hours of IPV training was strongly associated with perceived knowledge and actual knowledge, leading to improved attitudes and awareness toward IPV victims [56]. It is believed that both the quantity and quality of IPV training are critical factors to consider, as they can contribute to better perceived knowledge and actual knowledge. The current findings suggest the need for educational training to increase PHCPs’ knowledge, skills, and readiness for the identification and management of IPV.

At present, routine enquiry or screening for IPV is not the standard practice in primary care in Malaysia. There are also no standardized IPV guidelines or protocols available at the primary healthcare level. The current approach is limited to case findings, with selective IPV screening conducted for women attending maternity care visits or those presenting indicators of IPV. Previous studies have indicated that the availability of guidelines and protocols along with the provision of training are essential to improve providers’ readiness for IPV management [18, 38]. Without an established and explicit work process, PHCPs may perceive themselves or be perceived as unprepared to manage IPV cases. This study contributes to the understanding of the current level of readiness to respond to IPV among Malaysian PHCPs and its associated factors. The study’s findings emphasize the need for IPV training to equip PHCPs with the essential knowledge, skills, and positive attitudes necessary for effectively managing IPV. Establishing and implementing standardized IPV management guidelines in primary healthcare settings would further strengthen PHCPs’ role as first responders to provide immediate support and care for IPV victims.

### Recommendations

The current study’s findings lead to the recommendation of developing country-specific and culturally sensitive IPV training modules. These modules should cover the important aspects of identifying IPV and providing appropriate responses, including basic counselling skills, as PHCPs are often the first responders to psychological help for IPV victims. Additionally, PHCPs should be trained in trauma-informed care and the management of IPV-related injuries and mental health issues. Moreover, training programs should adopt a multidisciplinary approach, incorporating input from experts in IPV, mental health, social work, law enforcement, and community advocacy, to provide a holistic understanding of IPV and its impact on victims. IPV-related information should be updated consistently within healthcare organizations through the implementation of the Training of Trainers (ToT) framework. It is also recommended to incorporate IPV training into the medical/nursing curriculum while providers are in professional schools and continue IPV education in health care settings throughout their careers. This continuous education and training will empower PHCPs to respond effectively to IPV cases and provide compassionate care to patients affected by IPV.

In addition, health systems should prioritize the management of interpersonal violence alongside other major health concerns such as non-communicable diseases, by integrating efforts for early IPV identification, response and preventive strategies into routine primary care services. By recognizing IPV as a significant public health burden, healthcare systems can enhance their response capacity and contribute to reducing the negative physical and psychological impacts of violence on victims.

### Strengths and limitations

To our knowledge, this is the first cross-sectional study assessing the level of readiness to respond to IPV and its associated factors among PHCPs in Malaysia. A major strength of this study is the use of a multistage stratified cluster sampling design, which allows for a representative sample and enables generalizable findings to the target population of Malaysian PHCPs in public primary healthcare settings. Additionally, a satisfactory response rate of 64.7% was achieved, with a substantial sample of 1505 PHCPs from across all regions of the country responding to the survey. The study also used the psychometrically validated PREMIS-Malay questionnaire, which provides a reliable and accurate measure of Malaysian PHCPs’ readiness to manage IPV and facilitates comparisons of findings with international studies using the PREMIS tool.

Nevertheless, this study has several limitations. The cross-sectional design of the study limits the ability to establish cause-effect relationships between the studied variables. Another potential limitation is the possibility of social desirability bias from the self-reported data, given the sensitive nature of the topic. The study participants were mainly females. Female participants may have been more concerned about IPV issues, potentially influencing their responses, whereas male perspectives may not have been as thoroughly captured. Nevertheless, the gender imbalance aligns with the actual composition of the Malaysian primary healthcare workforce, where the majority of PHCPs, particularly nurses, are females. Therefore, the sample truly reflects the characteristics of the population studied.

### Conclusion and future directions

IPV is a complex public health issue that predominantly affects women’s health and well-being, and PHCPs are often the initial point of contact for patients experiencing IPV. The findings of this study revealed that most Malaysian PHCPs lack IPV-related training, have limited knowledge of IPV, and feel inadequately prepared to respond to IPV. The significant factors associated with greater perceived preparedness included longer work experience in primary care, prior IPV training, previous experience with IPV inquiry, and both good perceived and actual knowledge. These findings have meaningful implications for clinical practice and public health policy in Malaysia. The evidence from this study not only serves as baseline findings of PHCPs’ preparedness to manage IPV, but also guides future interventions and training programs to support PHCPs in responding effectively to IPV cases. Future efforts should focus on integrating IPV training into medical curricula, implementing structured continuous professional education, and developing standardized guidelines for IPV identification and management. A coordinated, multidisciplinary approach involving stakeholders, policymakers, and healthcare educators is needed to establish a supportive health system with clear referral pathways across all levels of care.

## Electronic supplementary material

Below is the link to the electronic supplementary material.

Supplementary Material 1

Supplementary Material 2

Supplementary Material 3

## Data Availability

The data that support the findings of this study are not publicly available due to the sensitive nature of the information of the respondents provided but are available from the corresponding author upon reasonable request.
