# Supplementary material for: Primary healthcare providers’ perceived preparedness to respond to intimate partner violence in the public primary healthcare setting: a cross-sectional study
Source: BMC Prim Care. 2025 Mar 31;26:92. doi: 10.1186/s12875-025-02793-2 (PMC11956219; doi:10.1186/s12875-025-02793-2)
Supplement: Supplementary file 1 — Supplementary Material 1 [file 12875_2025_2793_MOESM1_ESM.pdf]

KESEDIAAN PENGAMAL PERUBATAN UNTUK RESPON TEHADAP KEGANASAN PASANGAN INTIM DI PERINGKAT PENJAGAAN KESIHATAN PRIMER: TINJAUAN ATAS TALIAN

Jawapan jujur anda dalam tinjauan ini amat membantu dalam usaha kami mempertingkatkan kemahiran pengamal perubatan mengenalpasti dan mengendali kecederaan serta masalah kesihatan berkaitan **keganasan pasangan intim***. Sila nyatakan jawapan naluri pertama yang terlintas di fikiran anda, walaupun anda mungkin merasakan ianya tidak sesuai dari segi politik (*politically incorrect*). Jangan cuba memikirkan apa jawapan yang anda “patut” berikan. Semua respon akan dikodkan hanya menggunakan nombor, dirahsiakan, dan dianalisa secara berkumpulan untuk mengelakkan pendedahan maklumat peribadi.

Soal selidik ini mengandungi **enam (6) bahagian: (A) Profil Responden, (B) Latar Belakang, (C) Pengetahuan Sebenar, (D) Pendapat, (E), Isu Amalan, dan (F) Pengalaman Peribadi**. Sesetengah soalan mungkin kelihatan sama antara satu sama lain. Namun, kami memohon anda menjawab semua soalan bagi memastikan kebolehpercayaan penilaian. Terima kasih kerana meluangkan masa (dianggar selama 20 minit) untuk melengkapkan tinjauan ini.

*** Keganasan pasangan intim / *intimate partner violence* (IPV)**

- juga biasanya dikenali sebagai **keganasan rumah tangga/domestik (DV), keganasan pasangan atau keganasan keluarga.**
- biasanya melibatkan keganasan antara pasangan intim termasuk pasangan suami isteri atau teman lelaki/ wanita.

Bahagian A: Profil Responden

1. Umur: ________________

2. Jantina: [ ] Lelaki [ ] Perempuan

3. Alamat emel: ________________________ (Sila masukkan emel yang sah)

4a. Nombor telefon bimbit: ________________(Sila masukkan nombor sahaja, contoh: 012xxxxx88)

4b. Nombor telefon pejabat: ________________(Sila masukkan nombor sahaja, contoh: 03xxxx8888)

5. Bangsa:
   [ ] Melayu
   [ ] Cina
   [ ] India
   [ ] Bumiputera Sabah
   [ ] Bumiputera Sarawak
   [ ] Lain-lain, sila nyatakan ________________

6. Status perkahwinan:
   [ ] Tidak pernah berkahwin
   [ ] Berkahwin
   [ ] Berpisah
   [ ] Bercerai
   [ ] Janda/Duda
   [ ] Tinggal Bersama pasangan

7. Negeri anda bertugas: ______________________

8. Tempat bertugas (nama klinik): ______________________

9. Jawatan:
   - [ ] Pakar Perubatan Keluarga (FMS)
   - [ ] Pegawai Perubatan (MO)
   - [ ] Penolong Pegawai Perubatan (PPP)
   - [ ] Jururawat Terlatih (JT)
   - [ ] Jururawat Masyarakat (JM)

10. Tahap pendidikan tertinggi:
    - [ ] Sijil
    - [ ] Diploma
    - [ ] Ijazah Sarjana Muda
    - [ ] Ijazah Sarjana
    - [ ] Ijazah Doktor Falsafah/PhD

11. Berapa tahunkah anda telah bekerja di fasiliti kesihatan primer (termasuk Klinik Kesihatan, Klinik Desa, Klinik Komuniti (Klinik 1Malaysia), KKIA dan Klinik Bergerak) sehingga tahun terkini? ______ tahun
    (Nota: Jika kurang dari satu tahun, sila nyatakan jumlah tahun terdekat sebagai "1")

12. Bilangan purata pesakit yang anda rawat/kendalikan <u>dalam seminggu</u>: _____ pesakit
    (Nota: Sila nyatakan anggaran kasar bilangan purata pesakit)

13. Termasuk diri sendiri, berapa ramai pengamal perubatan di tempat kerja anda yang pernah menyertai kursus latihan keganasan pasangan intim <u>dalam tempoh 6 bulan yang lepas</u>?

    Ini mewakili :
    - [ ] Kesemua
    - [ ] Kebanyakan (melebihi separuh tetapi bukan semua)
    - [ ] Beberapa (melebihi sedikit tetapi kurang dari separuh)
    - [ ] Sedikit (tidak ramai tetapi melebihi seorang)
    - [ ] Tiada
    - [ ] Tidak tahu

Bahagian B: Latar Belakang

1. Berapa banyak latihan yang pernah anda ikuti/hadiri tentang isu keganasan pasangan intim sebelum ini?
   (*Boleh pilih lebih dari satu jawapan)*
   - [ ] Tiada
   - [ ] Telah membaca protokol institusi saya.
   - [ ] Tonton di TV/video/berita
   - [ ] Menghadiri ceramah atau syarahan
   - [ ] Menghadiri bengkel atau latihan kemahiran
   - [ ] Latihan dalam kelas di Institut Perubatan atau Kejururawatan /Kolej/ lain-lain
   - [ ] Latihan klinikal di Institut Perubatan atau Kejururawatan /Kolej/ lain-lain
   - [ ] Latihan residensi/fellowship/latihan pasca ijazah lain
   - [ ] Program Pendidikan Berterusan (CME/CNE)
   - [ ] Lain-lain latihan mendalam (melebihi 4 jam)
   - [ ] Lain-lain (nyatakan) ____

2. Anggaran jumlah jam keseluruhan latihan keganasan pasangan intim yang anda ikuti/hadiri sebelum ini:
   - [ ] Tiada
   - [ ] 1 – 5 jam
   - [ ] 6 – 15 jam
   - [ ] melebihi 15 jam

3. Sila pilih skala yang paling sesuai bagi menggambarkan tahap kesediaan anda melakukan tindakan berikut:
(1 = tidak bersedia; 2 = bersedia amat sedikit; 3 = bersedia sedikit; 4 = bersedia sederhana; 5 = agak bersedia; 6 = bersedia; 7 = sangat bersedia)

		Tidak Bersedia						*Sangat Bersedia*
a.	Bertanyakan soalan yang sesuai kepada pesakit tentang keganasan pasangan intim berdasarkan latar belakang, keadaan semasa dirawat dan situasi pesakit	1	2	3	4	5	6	7
b.	Memberikan respon yang sesuai kepada pesakit terhadap pendedahan keganasan	1	2	3	4	5	6	7
c.	Mengenalpasti indikator/petunjuk keganasan pasangan intim berdasarkan sejarah pesakit dan pemeriksaan fizikal	1	2	3	4	5	6	7
d.	Menilai kesediaan mangsa untuk berubah bagi mengatasi masalah keganasan pasangan intim	1	2	3	4	5	6	7
e.	Membantu mangsa keganasan pasangan intim menilai risiko mereka mengalami kecederaan maut	1	2	3	4	5	6	7
f.	Menilai keselamatan anak-anak mangsa keganasan pasangan intim	1	2	3	4	5	6	7
g.	Membantu mangsa keganasan pasangan intim membuat pelan keselamatan	1	2	3	4	5	6	7
h.	Mendokumentasikan sejarah keganasan pasangan intim dan hasil pemeriksaan fizikal dalam fail pesakit	1	2	3	4	5	6	7
i.	Membuat rujukan yang sesuai untuk keganasan pasangan intim	1	2	3	4	5	6	7

4. Sejauh manakah yang anda rasa anda tahu tentang:
(1 = langsung tidak tahu; 2 = amat sedikit; 3 = sedikit; 4 = sederhana; 5 = agak banyak; 6 = banyak; 7 = sangak banyak)

		langsung tidak tahu						*sangat banyak*
a.	Keperluan anda melaporkan keganasan pasangan intim mengikut undang-undang	1	2	3	4	5	6	7
b.	Tanda-tanda atau gejala keganasan pasangan intim	1	2	3	4	5	6	7
c.	Cara untuk mendokumentasikan keganasan pasangan intim dalam carta pesakit	1	2	3	4	5	6	7
d.	Sumber rujukan bagi mangsa keganasan pasangan intim	1	2	3	4	5	6	7
e.	Ciri-ciri pendera/pelaku keganasan pasangan intim	1	2	3	4	5	6	7
f.	Hubungkait antara keganasan pasangan intim dan kehamilan	1	2	3	4	5	6	7
g.	Kesan menyaksikan keganasan pasangan intim semasa zaman kanak-kanak	1	2	3	4	5	6	7
h.	Soalan-soalan yang perlu ditanyakan bagi mengenalpasti keganasan pasangan intim	1	2	3	4	5	6	7
i.	Sebab-sebab mangsa mungkin tidak mendedahkan keganasan pasangan intim	1	2	3	4	5	6	7
j.	Peranan anda dalam mengesan keganasan pasangan intim	1	2	3	4	5	6	7
k.	Apa yang boleh diperkatakan atau yang tidak boleh diperkatakan kepada seseorang pesakit dalam situasi keganasan pasangan intim	1	2	3	4	5	6	7
l.	Mengenalpasti keadaan bahaya bagi pesakit yang mengalami keganasan pasangan intim	1	2	3	4	5	6	7
m.	Membuat pelan keselamatan bersama dengan mangsa keganasan pasangan intim	1	2	3	4	5	6	7
n.	Peringkat-peringkat yang dialami seseorang mangsa keganasan pasangan intim dalam memahami dan mengubah situasi diri sendiri	1	2	3	4	5	6	7

Bahagian C: Pengetahuan Sebenar

Tanda satu jawapan bagi setiap perkara, melainkan dinyatakan sebaliknya.

1. Apakah faktor risiko yang <u>paling utama</u> bagi seseorang itu menjadi mangsa keganasan pasangan intim?

    [ ] Umur (<30tahun)
    [ ] Pasangan menyalahgunakan alkohol/dadah
    [ ] Jantina – perempuan
    [ ] Sejarah penderaan dalam keluarga
    [ ] Tidak tahu

2. Mana <u>satu</u> kenyataan berikut secara umumnya adalah benar tentang pendera?

    [ ] Mereka mempunyai masalah mengawal kemarahan.
    [ ] Mereka menggunakan keganasan sebagai cara untuk mengawal pasangan mereka.
    [ ] Mereka bertindak ganas akibat minum arak atau menggunakan dadah.
    [ ] Mereka akan bergaduh dengan sesiapa sahaja.

3. Manakah di antara berikut merupakan tanda amaran bahawa seseorang pesakit mungkin telah didera oleh pasangannya? (Boleh pilih lebih dari satu jawapan)

    [ ] Sakit kronik yang tidak dapat dijelaskan
    [ ] Keresahan
    [ ] Penyalahgunaan substans
    [ ] Kerap cedera
    [ ] Kemurungan

4. Manakah di antara berikut adalah sebab seseorang mangsa keganasan pasangan intim mungkin tidak mampu meninggalkan hubungan keganasan dengan pendera?
   (Boleh pilih lebih dari satu jawapan)

    [ ] Takut akan pembalasan (hukuman oleh pendera)
    [ ] Kebergantungan kewangan kepada pendera
    [ ] Kepercayaan budaya/agama
    [ ] Keperluan anak-anak
    [ ] Rasa cinta terhadap pasangan
    [ ] Isolasi/rasa terasing

5. Manakah di antara berikut merupakan cara yang paling sesuai untuk bertanya tentang keganasan pasangan intim? (Boleh pilih lebih dari satu jawapan)

    [ ] "Adakah anda mangsa keganasan pasangan intim?"
    [ ] "Pernahkah pasangan anda menyakiti atau mengancam anda?"
    [ ] "Pernahkah anda berasa takut terhadap pasangan anda?"
    [ ] "Pernahkan pasangan anda memukul atau mencederakan anda?"

6. Manakah di antara berikut secara umumnya benar? (Boleh pilih lebih dari satu jawapan)

    [ ] Terdapat tanda-tanda lazim (selain dari kecederaan fizikal) yang ada pada pesakit didera.
    [ ] Terdapat corak tingkah laku pada pasangan-pasangan yang mungkin petanda keganasan pasangan intim.
    [ ] Terdapat bahagian badan tertentu yang sering menjadi sasaran dalam kes-kes keganasan pasangan intim.
    [ ] Terdapat corak kecederaan yang sering dikaitkan dengan keganasan pasangan intim.
    [ ] Kecederaan pelbagai peringkat penyembuhan mungkin menunjukkan penderaan.

7. Adakah anda mempunyai sebarang pengetahuan mengenai Peringkat-peringkat Perubahan (*The Stages of Change*)?
   [ ] Ya, sila ke soalan 8
   [ ] Tidak, sila langkau ke soalan 9

8. Sila padankan penerangan mengenai tingkah laku dan perasaan pesakit yang mempunyai sejarah keganasan pasangan intim dengan peringkat perubahan yang sesuai seperti berikut:

   1 = Pra-kontemplasi (*Pre-contemplation*)
   2 = Kontemplasi (*Contemplation*)
   3 = Persediaan (*Preparation*)
   4 = Bertindak (*Action*)
   5 = Penerusan *(Maintenance)*
   6 = Penamatan (*Termination*)

	1	2	3	4	5	6
a. Mula membuat perancangan untuk meninggalkan pasangan	○	○	○	○	○	○
b. Menafikan wujudnya masalah	○	○	○	○	○	○
c. Mula berfikir bahawa penderaan bukan salah diri sendiri	○	○	○	○	○	○
d. Terus mengamalkan perubahan tingkahlaku	○	○	○	○	○	○
e. Mendapatkan perintah perlindungan	○	○	○	○	○	○

9. Pilih **B** untuk "betul", **S** untuk "salah", atau **TT** jika "tidak tahu" untuk jawapan berikut:

a.	Penggunaan alkohol adalah peramal *(predictor)* yang paling utama kepada kejadian keganasan pasangan intim.	B	S	TT
b.	Mangsa tidak mempunyai alasan yang kukuh untuk kekal dalam suatu perhubungan penderaan.	B	S	TT
c.	Sebab-sebab mengesyaki keganasan pasangan intim tidak patut dinyatakan dalam carta pesakit sekiranya pesakit tidak mendedahkan keganasan tersebut.	B	S	TT
d.	Apabila bertanya kepada pesakit tentang keganasan pasangan intim, pengamal perubatan patut menggunakan perkataan "didera" atau "dipukul."	B	S	TT
e.	Menyokong keputusan pesakit untuk meneruskan perhubungan walaupun keganasan berlaku akan membiarkan penderaan itu berterusan.	B	S	TT
f.	Mangsa keganasan pasangan intim dapat membuat pilihan yan sesuai mengenai cara mengendalikan situasi mereka.	B	S	TT
g.	Pengamal perubatan tidak patut memaksa pesakit untuk mengakui bahawa mereka hidup dalam perhubungan yang bersifat penderaan.	B	S	TT
h.	Mangsa keganasan pasangan intim lebih berisiko mengalami kecederaan apabila mereka meninggalkan perhubungan tersebut.	B	S	TT
i.	Kecederaan dicekik jarang berlaku dalam kes-kes keganasan pasangan intim.	B	S	TT
j.	Membenarkan pasangan atau rakan hadir bersama sewaktu pengambilan sejarah dan pemeriksaan fizikal dapat memastikan keselamatan mangsa keganasan pasangan intim.	B	S	TT
k.	Pengamal perubatan diberi mandat untuk melaporkan kejadian kanak-kanak yang menyaksikan keganasan pasangan intim kepada Perkhidmatan Perlindungan Kanak-kanak, walaupun kanak-kanak itu tidak berada dalam keadaan yang jelas berbahaya.	B	S	TT

Bahagian D: Pendapat

Bagi setiap kenyataan berikut, sila tandakan respon anda menurut skala dari "Sangat tidak setuju" (1) hingga "Sangat setuju" (7). Sila ambil perhatian bahawa bagi soalan berbentuk pendapat, tiada respon yang betul atau salah. Sila jawab semua soalan dengan teliti dan sejujur mungkin.

Kenyataan	***Sangat Tidak setuju***		***Tidak setuju***		***Setuju***		***Sangat setuju***
1. Sekiranya mangsa keganasan pasangan intim tidak mengakui ada penderaaan, tidak banyak yang boleh saya lakukan untuk membantu.	1	2	3	4	5	6	7
2. Saya bertanya kepada semua pesakit baru mengenai penderaan dalam perhubungan mereka.	1	2	3	4	5	6	7
3. Saya mampu mengenalpasti keganasan pasangan intim tanpa bertanya kepada pesakit mengenainya.	1	2	3	4	5	6	7
4. Saya tidak mempunyai latihan yang mencukupi untuk membantu individu menangani situasi keganasan pasangan intim.	1	2	3	4	5	6	7
5. Pesakit yang menyalahgunakan alkohol atau dadah berkemungkinan mempunyai sejarah keganasan pasangan intim.	1	2	3	4	5	6	7
6. Saya berasa selesa berbincang mengenai keganasan pasangan intim bersama pesakit saya.	1	2	3	4	5	6	7
7. Saya tidak mempunyai kemahiran yang diperlukan untuk berbincang mengenai penderaan dengan seseorang mangsa keganasan pasangan intim:							
a) Perempuan	1	2	3	4	5	6	7
b) Lelaki	1	2	3	4	5	6	7
c) dari latar belakang budaya/etnik/agama berlainan	1	2	3	4	5	6	7
8. Saya sedar akan keperluan undang-undang dinegara ini mengenai pelaporan kes-kes yang disyaki							
a) keganasan pasangan intim	1	2	3	4	5	6	7
b) penderaan kanak-kanak	1	2	3	4	5	6	7
c) penderaan warga emas	1	2	3	4	5	6	7
9. Pengamal perubatan tiada masa untuk membantu pesakit menangani keganasan pasangan intim.	1	2	3	4	5	6	7
10. Saya mampu mengumpul maklumat yang diperlukan untuk mengenalpasti keganasan pasangan intim sebagai punca penyakit (cth., kemurungan, migrain).	1	2	3	4	5	6	7

Kenyataan	***Sangat Tidak setuju***		***Tidak setuju***		***Setuju***		***Sangat setuju***
11. Sekiranya pesakit enggan berbincang mengenai penderaan, pengamal perubatan hanya boleh merawat kecederaan pesakit.	1	2	3	4	5	6	7
12. Tempat kerja saya memberikan saya masa yang cukup untuk respon kepada mangsa keganasan pasangan intim.	1	2	3	4	5	6	7
13. Saya dapat menghubungi perkhidmatan dalam komuniti bagi mewujudkan rujukan untuk mangsa keganasan pasangan intim.	1	2	3	4	5	6	7
14. Penyalahgunaan alkohol adalah punca utama keganasan pasangan intim.	1	2	3	4	5	6	7
15. Saya terlalu sibuk untuk menyertai pasukan pelbagai disiplin yang menguruskan kes keganasan pasangan intim.	1	2	3	4	5	6	7
16. Saringan keganasan pasangan intim berkemungkinan menyinggung perasaan mereka yang disaring.	1	2	3	4	5	6	7
17. Terdapat ruang khusus yang mencukupi untuk saya memberikan penjagaan kepada mangsa keganasan pasangan intim.	1	2	3	4	5	6	7
18. Saya mampu mengumpul maklumat yang diperlukan untuk mengenalpasti keganasan pasangan intim sebagai punca kecederaan pesakit (cth., lebam, patah tulang, dsb.)	1	2	3	4	5	6	7
19. Penggunaan alkohol atau dadah adalah berkaitan dengan keganasan pasangan intim.	1	2	3	4	5	6	7
20. Tahap sekuriti di tempat kerja saya adalah mencukupi bagi membolehkan perbincangan mengenai penderaan dengan pesakit dilakukan dengan selamat.	1	2	3	4	5	6	7
21. Saya perlu menumpukan perhatian saya kepada masalah kesihatan lain yang lebih tinggi prioriti.	1	2	3	4	5	6	7

Bahagian E: Isu Amalan

Untuk soalan-soalan berikut, sila pilih jawapan berpadanan dengan amalan klinikal anda berkaitan dengan mengenalpasti dan memberi respon kepada pesakit atau wanita mangsa keganasan pasangan intim.

1. Dalam tempoh 6 bulan lepas, berapakah anggaran kes keganasan pasangan intim yang telah anda kenalpasti (termasuk mengenalpasti kes akut, kes penderaan yang masih berterusan, atau pesakit mendedahkan sejarah penderaan lalu)?
   - [ ] Tiada
   - [ ] 1-5
   - [ ] 6-10
   - [ ] 11-20
   - [ ] 21 atau lebih

2. Pernahkah anda bertanya kepada pesakit tentang keganasan pasangan intim?
   - [ ] Ya, sila ke soalan 3
   - [ ] Tidak, sila langkau ke soalan 4

3. Sila tandakan situasi pesakit di mana anda bertanya tentang keganasan pasangan intim.
   (Boleh pilih lebih daripada satu jawapan)

   - [ ] Saya bertanya kepada semua pesakit baharu
   - [ ] Saya bertanya kepada semua pesakit wanita baharu
   - [ ] Saya bertanya kepada semua pesakit yang ada indikator penderaan dari sejarah atau pemeriksaan fizikal
   - [ ] Saya bertanya kepada semua pesakit 7amper semasa pemeriksaan tahunan mereka
   - [ ] Saya bertanya kepada semua pesakit hamil pada waktu tertentu kehamilan mereka
   - [ ] Saya bertanya kepada semua pesakit secara berkala
   - [ ] Saya bertanya kepada semua pesakit 7amper secara berkala
   - [ ] Saya bertanya kepada pesakit kategori tertentu sahaja *(tanda di bawah)*
     - [ ] Remaja
     - [ ] Wanita muda dewasa (bawah 30 tahun)
     - [ ] Wanita warga emas (melebihi 60 tahun)
     - [ ] Wanita bujang atau telah bercerai
     - [ ] Wanita berkahwin
     - [ ] Wanita yang bermasalah penyalahgunaan 7amper7 atau bahan lain
     - [ ] Ibu tunggal
     - [ ] Wanita imigran
     - [ ] Wanita lesbian
     - [ ] Lelaki homoseksual
     - [ ] Wanita kemurungan / berniat bunuh diri
     - [ ] Wanita mengandung
     - [ ] Ibu kepada semua pesakit kanak-kanak saya (jika berkenaan)
     - [ ] Ibu kepada pesakit kanak-kanak yang menunjukkan tanda-tanda pernah menyaksikan keganasan pasangan intim
     - [ ] Ibu kepada kanak-kanak yang disahkan atau disyaki mangsa penderaan, pengabaian
     - [ ] Lain-lain. Sila nyatakan: ____

4. Dalam tempoh 6 bulan lepas, berapa kerapkah anda bertanya tentang kemungkinan keganasan pasangan intim apabila anda berjumpa pesakit dengan gejala berikut:

		Tidak pernah	*Jarang*	*kadang-kala*	*Hampir selalu*	*Selalu*	*T/B*
a.	Kecederaan	1	2	3	4	5	6
b.	Sakit pelvis kronik	1	2	3	4	5	6
c.	Sindrom usus merengsa (*Irritable bowel syndrome)*	1	2	3	4	5	6
d.	Sakit kepala	1	2	3	4	5	6
e.	Kemurungan/Keresahan	1	2	3	4	5	6
f.	Tekanan darah tinggi	1	2	3	4	5	6
g.	Gangguan pemakanan	1	2	3	4	5	6

5. Dalam tempoh 6 bulan yang lepas, manakah antara tindakan berikut yang telah anda ambil apabila anda mengenalpasti keganasan pasangan intim atau apabila pesakit mendedahkan keganasan pasangan intim? (Boleh pilih lebih dari satu jawapan)

[ ] Tidak mengenalpasti keganasan pasangan intim dalam tempoh 6 bulan yang lepas
[ ] Memberikan maklumat (nombor telefon, risalah, maklumat lain) kepada pesakit
[ ] Menasihati pesakit tentang pilihan yang mungkin mereka ada.
[ ] Menilai keselamatan mangsa
[ ] Menilai keselamatan anak mangsa
[ ] Membantu pesakit membuat pelan keselamatan diri
[ ] Merujuk pesakit ke:
   [ ] Pusat Krisis Bersepadu (*One Stop Crisis Centre)*
   [ ] Perkhidmatan Perlindungan Kanak-kanak (*Child Protective Services*)
   [ ] Terapi individu/pasangan
   [ ] Advokat undang-undang / Advokat saksi mangsa
   [ ] Terapi /kumpulan sokongan kanak-kanak
   [ ] Program rawatan pendera
   [ ] Pekerja sosial/pegawai kebajikan masyarakat/advokat setempat
   [ ] Pemimpin/pertubuhan agama
   [ ] Program/tempat perlindungan wanita didera
   [ ] Kumpulan sokongan wanita didera
   [ ] Kaunseling penyalahgunaan alkohol/substans
   [ ] Talian Hotline Keganasan Domestik/Rumah Tangga Kebangsaan
   [ ] Talian Hotline Keganasan Domestik/Rumah Tangga Tempatan
   [ ] Polis atau penguatkuasa undang-undang tempatan
   [ ] Bantuan perumahan, pendidikan, pekerjaan atau kewangan.
   [ ] Rujukan lain (terangkan): ____
   [ ] Tindakan lain (terangkan): ____

6. Adakah terdapat protokol pengurusan mangsa dewasa keganasan pasangan intim di klinik/tempat kerja anda? (Sila pilih satu jawapan sahaja)
[ ] Ya, dan sering digunakan
[ ] Ya, dan digunakan sedikit sebanyak
[ ] Ya, tetapi tidak digunakan
[ ] Tidak
[ ] Tidak pasti

7. Adakah anda tahu tentang polisi Kementerian Kesihatan Malaysia mengenai pengendalian kes keganasan pasangan intim?
[ ] Ya
[ ] Tidak

8. Adakah kamera tersedia untuk mengambil gambar kecederaan mangsa keganasan pasangan intim di tempat kerja anda?
   - [ ] Ya - - Jenis: [ ] Polaroid or kamera instant lain, [ ] Digital, [ ] lain-lain: _____________
   - [ ] Tidak
   - [ ] Tidak pasti

9. Adakah perundangan di negara anda telah memberi mandat supaya melaporkan kes keganasan pasangan intim melibatkan orang dewasa yang kompeten/tidak rentan *(non-vulnerable)?*
   - [ ] Ya
   - [ ] Tidak
   - [ ] Tidak pasti

10. Untuk setiap mangsa keganasan pasangan intim yang telah anda kenalpasti <u>dalam tempoh 6 bulan lepas</u>, berapa kerapkah anda telah:

	Tidak pernah	*Jarang*	*kadang-kala*	*Hampir selalu*	*Selalu*	*T/B*
a. Mendokumen kenyataan pesakit mengenai keganasan pasangan intim dalam carta	1	2	3	4	5	6
b. Menggunakan peta badan untuk mendokumentasikan kecederaan pesakit	1	2	3	4	5	6
c. Mengambil foto kecederaan mangsa untuk disertakan dalam carta	1	2	3	4	5	6
d. Melaporkan kepada pihak berkuasa berkenaan walaupun tidak diberi mandat	1	2	3	4	5	6
e. Menilai keselamatan mangsa keganasan pasangan intim	1	2	3	4	5	6
f. Menilai keselamatan anak mangsa keganasan pasangan intim	1	2	3	4	5	6
g. Membantu mangsa keganasan pasangan intim membuat pelan keselamatan	1	2	3	4	5	6
h. Menghubungi agensi/individu yang memberi perkhidmatan menangani keganasan pasangan intim	1	2	3	4	5	6
i. Memberi kenyataan yang mengesahkan pengalaman didera / sokongan	1	2	3	4	5	6
j. Menyediakan maklumat asas mengenai keganasan pasangan intim	1	2	3	4	5	6
k. Menyediakan rujukan dan/atau sumber maklumat	1	2	3	4	5	6

11. Adakah bahan pendidikan pesakit atau sumber maklumat berkaitan keganasan pasangan intim (poster, brosur, dll.) tersedia di tempat kerja anda? (Sila pilih satu jawapan sahaja)
   - [ ] Ya, dipaparkan dengan baik, dan diakses oleh pesakit
   - [ ] Ya, dipaparkan dengan baik, tetapi tidak boleh diakses oleh pesakit
   - [ ] Ya, tetapi tidak dipaparkan dengan baik
   - [ ] Tidak
   - [ ] Tidak pasti

12. Adakah anda menyediakan bahan pendidikan atau sumber berkaitan keganasan pasangan intim untuk pesakit yang didera? (Sila pilih satu jawapan sahaja)

    [ ] Ya, hampir selalu
    [ ] Ya, sekira ia selamat untuk pesakit
    [ ] Ya, tetapi hanya atas permintaan pesakit
    [ ] Tidak, kerana sumber rujukan dalam komuniti adalah tidak mencukupi
    [ ] Tidak, kerana secara umumnya saya tidak merasakan bahan-bahan ini adalah berguna
    [ ] Tidak, sebab lain (sila nyatakan) ________________________

13. Adakah anda rasa sumber rujukan untuk pesakit dewasa keganasan pasangan intim **di tempat kerja anda** adalah mencukupi (termasuk rujukan kesihatan mental)?

    [ ] Ya
    [ ] Tidak
    [ ] Tidak pasti

14. Adakah anda rasa anda mempunyai pengetahuan mencukupi mengenai sumber rujukan untuk pesakit **dalam komuniti** (termasuk pusat perlindungan or kumpulan sokongan) bagi mangsa dewasa keganasan pasangan intim?

    [ ] Ya
    [ ] Tidak
    [ ] Tidak pasti

Bahagian F: Pengalaman Peribadi

1. Pernahkah anda mengalami keganasan fizikal, penderaan seksual, penderaan emosi, intimidasi, kekangan ekonomi atau ancaman keganasan dalam hubungan pasangan intim?

    [ ] Ya
    [ ] Tidak

2. Pernahkah anda menyaksikan sebarang keganasan fizikal, penderaan seksual atau psikologi terhadap ahli keluarga anda?

    [ ] Ya
    [ ] Tidak

Terima kasih kerana melengkapkan tinjauan ini.
