# Supplementary material for: Primary healthcare providers’ perceived preparedness to respond to intimate partner violence in the public primary healthcare setting: a cross-sectional study
Source: BMC Prim Care. 2025 Mar 31;26:92. doi: 10.1186/s12875-025-02793-2 (PMC11956219; doi:10.1186/s12875-025-02793-2)
Supplement: Supplementary file 2 — Supplementary Material 2 [file 12875_2025_2793_MOESM2_ESM.pdf]

PRIMARY HEALTHCARE PROVIDERS' READINESS TO RESPOND TO INTIMATE PARTNER VIOLENCE: AN ONLINE SURVEY

Your candid responses on the following survey will greatly assist us in our attempt to improve healthcare providers' recognition and management of **intimate partner violence (IPV)***-related injuries and illnesses. Please record your first, instinctive answer, even if you don't think it is "politically correct." Don't try to think about what your answers "should" be. All responses will be coded by an identifying number only, kept confidential, and analyzed in group form so that no personal information is revealed.

This survey contains **six (6) sections: (A) Respondent Profile, (B) Background, (C) Actual Knowledge, (D) Opinions, (E) Practice Issues, and (F) Personal Experience**. Some questions may seem similar to others. However, we ask that you answer all questions to help ensure the reliability of the assessment. Thank you for taking the time (approximately 20 minutes) to complete this survey.

* Intimate partner violence (IPV) is also commonly referred to as domestic violence (DV), partner violence, or family violence. It is typically violence between intimate partners including spouses or boy/girlfriends.

Section A: Respondent Profile

1. Your Age: ____________ years

2. Gender: [ ] Male [ ] Female

3. Email address: ______________________ (Please enter a valid email address)

4a. Mobile phone number: _________________(Please enter the number only, example: 012xxxxx88)

4b. Office phone number: _________________(Please enter the number only, example: 03xxxx8888)

5. Ethnicity:
   - [ ] Malay
   - [ ] Chinese
   - [ ] Indian
   - [ ] Bumiputera of Sabah
   - [ ] Bumiputera of Sarawak
   - [ ] Others, please specify __________________

6. Marital status:
   - [ ] Never married
   - [ ] Married
   - [ ] Separated
   - [ ] Divorcee
   - [ ] Widow/Widower
   - [ ] Living with partner

7. State in which you practice: ________________________

8. Place of duty (name of clinic): ______________________

9. Position:
   - [ ] Family medicine specialist (FMS)
   - [ ] Medical officer (MO)
   - [ ] Assistant medical officer (AMO)
   - [ ] Staff nurse (SN)
   - [ ] Community nurse (CN)

10. Highest education level:
    - [ ] Certified
    - [ ] Diploma
    - [ ] Bachelor
    - [ ] Master
    - [ ] PhD / Doctorate

11. How long have you been working in a health clinic to the recent year? ______ year
    (Note: if less than one year, please provide the nearest number of years as "1")

12. Average number of patients you care for per week: _____ patients (Note: please provide a rough estimate)

13. Including yourself, how many practitioners at your workplace have participated in an intimate partner violence (IPV) training course <u>in the past 6 months</u>?

    This represents:
    - [ ] All
    - [ ] Most (More than half but not all)
    - [ ] Some (More than a few but less than a half)
    - [ ] A few (Not many but more than one)
    - [ ] None
    - [ ] Don't know

Section B: Background

1. How much previous training about intimate partner violence (IPV) issues have you had?
   (Please check all that apply.)
   - [ ] None
   - [ ] Read my institution's protocol
   - [ ] Watched a TV/video/news
   - [ ] Attended a lecture or talk
   - [ ] Attended a skills-based training or workshop
   - [ ] Medical/nursing/other school—classroom training
   - [ ] Medical/nursing/other school—clinical setting
   - [ ] Residency/fellowship/other post grad training
   - [ ] CME/CNE program
   - [ ] Other in-depth training (more than 4 hours)
   - [ ] Other (specify) ____

2. Estimated total number of hours of previous IPV training:
   - [ ] None
   - [ ] 1 – 5 hours
   - [ ] 6 – 15 hours
   - [ ] More than 15 hours

3. Please choose the number which best describes how prepared you feel to perform the following:
(1 = Not prepared; 2 = Minimally prepared; 3 = Slighty prepared; 4 = Moderately prepared; 5 = Fairly well prepared; 6 = Well prepared; 7 = Quite well prepared)

		Not Prepared 1	2	3	4	5	6	Quite Well Prepared 7
a.	Ask appropriate* questions about IPV (*asking questions appropriately based on patient's background, presenting conditions and circumstances)	1	2	3	4	5	6	7
b.	Appropriately respond to disclosures of abuse	1	2	3	4	5	6	7
c.	Identify IPV indicators based on patient history, and physical examination	1	2	3	4	5	6	7
d.	Assess an IPV victim's readiness to change* (*readiness to change to solve the problem)	1	2	3	4	5	6	7
e.	Help an IPV victim assess his/her danger of lethality	1	2	3	4	5	6	7
f.	Conduct a safety assessment for the victim's children	1	2	3	4	5	6	7
g.	Help an IPV victim create a safety plan	1	2	3	4	5	6	7
h.	Document IPV history and physical examination findings in patient's file	1	2	3	4	5	6	7
i.	Make appropriate referrals for IPV	1	2	3	4	5	6	7

4. How much do you feel you now know about:
(1 = Nothing; 2 = Very little; 3 = A little; 4 = A moderata amount; 5 = A fair amount; 6 = Quite a bit;
7 = Very much

		Nothing 1	2	3	4	5	6	Very much 7
a.	Your legal reporting requirements for IPV	1	2	3	4	5	6	7
b.	Signs or symptoms of IPV	1	2	3	4	5	6	7
c.	How to document IPV in patient's chart	1	2	3	4	5	6	7
d.	Referral sources for IPV victims	1	2	3	4	5	6	7
e.	Perpetrators of IPV	1	2	3	4	5	6	7
f.	Relationship between IPV and pregnancy	1	2	3	4	5	6	7
g.	Recognizing the childhood effects of witnessing IPV	1	2	3	4	5	6	7
h.	What questions to ask to identify IPV	1	2	3	4	5	6	7
i.	Why a victim might not disclose IPV	1	2	3	4	5	6	7
j.	Your role in detecting IPV	1	2	3	4	5	6	7
k.	What to say or not to say to a patient in IPV situations	1	2	3	4	5	6	7
l.	Determining danger for a patient experiencing IPV	1	2	3	4	5	6	7
m.	Developing a safety plan with an IPV victim	1	2	3	4	5	6	7
n.	The stages an IPV victim experiences in understanding and changing his/her situation	1	2	3	4	5	6	7

Section C: Actual Knowledge

Check one answer per item, unless noted otherwise.

1. What is the strongest single risk factor for becoming a victim of intimate partner violence?

    [ ] Age (<30yrs)
    [ ] Partner abuses alcohol/drugs
    [ ] Gender – female
    [ ] Family history of abuse
    [ ] Don't know

2. Which one of the following is generally true about batterers?

    [ ] They have trouble controlling their anger
    [ ] They use violence as a means of controlling their partners
    [ ] They are violent because they drink or use drugs
    [ ] They pick fights with anyone

3. Which of the following are warning signs that a patient may have been abused by his/her partner? (check all that apply)

    [ ] Chronic unexplained pain
    [ ] Anxiety
    [ ] Substance abuse
    [ ] Frequent injuries
    [ ] Depression

4. Which of the following are reasons an IPV victim may not be able to leave a violent relationship? (check all that apply)

    [ ] Fear of retribution (punishment by perpetrator)
    [ ] Financial dependence on the perpetrator
    [ ] Religious/cultural beliefs
    [ ] Children's needs
    [ ] Love for one's partner
    [ ] Isolation

5. Which of the following are the most appropriate ways to ask about IPV? (check all that apply)

    [ ] "Are you a victim of intimate partner violence?
    [ ] "Has your partner ever hurt or threatened you?"
    [ ] "Have you ever been afraid of your partner?"
    [ ] "Has your partner ever hit or hurt you?"

6 . Which of the following is/are generally true? (check all that apply)

    [ ] There are common, non-injury presentations of abused patients
    [ ] There are behavioral patterns in couples that may indicate IPV
    [ ] Specific areas of the body are most often targeted in IPV cases
    [ ] There are common injury patterns associated with IPV
    [ ] Injuries in different stages of recovery may indicate abuse

7. Do you have any knowledge of The Stages of Change?

   [ ] Yes, please go to question 8
   [ ] No, please skip to question 9

8. Please match the following descriptions of the behaviours and feelings of patients with a history of IPV with the appropriate stage of change.

   1 = Pre-contemplation   2 = Contemplation   3 = Preparation
   4 = Action   5 = Maintenance   6 = Termination

	1	2	3	4	5	6
a. Begins making plans for leaving the abusive partner	○	○	○	○	○	○
b. Denies there's a problem	○	○	○	○	○	○
c. Begins thinking the abuse is not their own fault	○	○	○	○	○	○
d. Continues changing behaviors	○	○	○	○	○	○
e. Obtains order(s) for protection	○	○	○	○	○	○

9. Choose **T** for "true", **F** for "false", or **DK** if you "don't know" the answer to the following:

a.	Alcohol consumption is the greatest single predictor of the likelihood of IPV.	T	F	DK
b.	There are no good reasons for not leaving an abusive relationship.	T	F	DK
c.	Reasons for concern about IPV should not be included in a patient's chart if s/he does not disclose the violence.	T	F	DK
d.	When asking patients about IPV, healthcare providers should use the words "abused" or "battered."	T	F	DK
e.	Being supportive of a patient's choice to remain in a violent relationship would condone the abuse.	T	F	DK
f.	Victims of IPV are able to make appropriate choices about how to handle their situation.	T	F	DK
g.	Healthcare providers should not pressure patients to acknowledge that they are living in an abusive relationship.	T	F	DK
h.	Victims of IPV are at greater risk of injury when they leave the relationship.	T	F	DK
i.	Strangulation injuries are rare in cases of IPV.	T	F	DK
j.	Allowing partners or friends to be present during a patient's history and physical exam ensures safety for an IPV victim.	T	F	DK
k.	Even if the child is not in immediate danger, healthcare providers are mandated to report an instance of a child witnessing IPV to Child Protective Services.	T	F	DK

Section D: Opinions

For each of the following statements, please indicate your response on the scale from "Strongly Disagree" (1) to "Strongly Agree" (7). Kindly note that there are no right or wrong answers for opinion-based questions. Please answer all questions as thoughtfully and honestly as possible.

Statements	Strongly Disagree		Disagree		Agree		Strongly Agree
1. If an IPV victim does not acknowledge the abuse, there is very little that I can do to help.	1	2	3	4	5	6	7
2. I ask all new patients about abuse in their relationships.	1	2	3	4	5	6	7
3. I am capable of identifying IPV without asking my patient about it.	1	2	3	4	5	6	7
4. I do not have sufficient training to assist individuals in addressing situations of IPV.	1	2	3	4	5	6	7
5. Patients who abuse alcohol or other drugs are likely to have a history of IPV.	1	2	3	4	5	6	7
6. I feel comfortable discussing IPV with my patients.	1	2	3	4	5	6	7
7. I don't have the necessary skills to discuss abuse with an IPV victim who is:							
a) Female	1	2	3	4	5	6	7
b) Male	1	2	3	4	5	6	7
c) from a different cultural/ethnic/religious background	1	2	3	4	5	6	7
8. I am aware of legal requirements in this country regarding reporting of suspected cases of							
a) IPV	1	2	3	4	5	6	7
b) child abuse	1	2	3	4	5	6	7
c) elder abuse	1	2	3	4	5	6	7
9. Healthcare providers do not have the time to assist patients in addressing IPV.	1	2	3	4	5	6	7
10. I am able to gather the necessary information to identify IPV as the underlying cause of patient illnesses (e.g., depression, migraines).	1	2	3	4	5	6	7
11. If a patient refuses to discuss the abuse, healthcare providers can only treat the patient's injuries.	1	2	3	4	5	6	7
12. My practice setting allows me adequate time to respond to victims of IPV.	1	2	3	4	5	6	7
13. I have contacted services within the community to establish referrals for IPV victims.	1	2	3	4	5	6	7

Statements	Strongly Disagree 1	2	Disagree 3	4	Agree 5	6	Strongly Agree 7
14. Alcohol abuse is a leading cause of IPV.	1	2	3	4	5	6	7
15. I am too busy to participate on a multidisciplinary team that manages IPV cases.	1	2	3	4	5	6	7
16. Screening for IPV is likely to offend those who are screened.	1	2	3	4	5	6	7
17. There is adequate private space for me to provide care for victims of IPV.	1	2	3	4	5	6	7
18. I am able to gather the necessary information to identify IPV as the underlying cause of patient injuries (e.g., bruises, fractures, etc.).	1	2	3	4	5	6	7
19. Use of alcohol or other drugs is related to IPV victimization.	1	2	3	4	5	6	7
20. There is adequate security at my workplace to safely permit discussion of abuse with patients.	1	2	3	4	5	6	7
21. I need to focus my attention on other health problems that have a higher priority.	1	2	3	4	5	6	7

Section E: Practice Issues

Please answer the following questions that match your clinical practice related to identifying and responding of patients or women who are victims of intimate partner violence (IPV).

1. How many cases or disclosure of IPV (including picked up an acute case, uncovered ongoing abuse, or had a patient disclose a past history) would you estimate you have encountered in the past 6 months?
   [ ] None
   [ ] 1-5
   [ ] 6-10
   [ ] 11-20
   [ ] 21 or more

2. Have you ever asked patients about IPV?
   [ ] Yes, please go to question 3
   [ ] No, please skip to question 4

3. Please indicate the patient situation in which you screen or ask about IPV. (check all that apply)
   [ ] I ask all new patients
   [ ] I ask all new female patients
   [ ] I ask all patients with abuse indicators on history or exam
   [ ] I ask all female patients at the time of their annual exam
   [ ] I ask all pregnant patients at specific times of their pregnancy
   [ ] I ask all patients periodically
   [ ] I ask all female patients periodically
   [ ] I ask certain patient categories only (check below)
      [ ] Teenagers
      [ ] Young adult women (under 30 years old)
      [ ] Elderly women (over 60 years old)
      [ ] Single or divorced women
      [ ] Married women
      [ ] Women with alcohol or other substance abuse
      [ ] Single mothers
      [ ] Immigrant women
      [ ] Lesbian women
      [ ] Homosexual men
      [ ] Depressed/suicidal women
      [ ] Pregnant women
      [ ] Mothers of all my pediatric patients (if applicable)
      [ ] Mothers of pediatric patients who show signs of witnessing IPV
      [ ] Mothers of children with confirmed or suspected child abuse, neglect
      [ ] Other. Please specify: _________

4. How often in the past six months have you asked about the possibility of IPV when seeing patients with the following:

		Never	Seldom	Some-times	Nearly always	Always	N/A
a.	Injuries	1	2	3	4	5	6
b.	Chronic pelvic pain	1	2	3	4	5	6
c.	Irritable bowel syndrome	1	2	3	4	5	6
d.	Headaches	1	2	3	4	5	6
e.	Depression/Anxiety	1	2	3	4	5	6
f.	Hypertension	1	2	3	4	5	6
g.	Eating disorders	1	2	3	4	5	6

5. In the past 6 months, which of the following actions have you taken when you identified IPV or when patient disclosed IPV? (check all that apply)

   [ ] Have not identified IPV in the past 6 months
   [ ] Provided information (phone numbers, pamphlets, other information) to patient
   [ ] Counseled patient about options she / he may have
   [ ] Conducted a safety assessment for the victim
   [ ] Conducted a safety assessment for victim's children
   [ ] Helped patient develop a personal safety plan
   [ ] Referred patient to:
      [ ] One Stop Crisis Centre
      [ ] Child Protective Services
      [ ] Individual/couples therapy
      [ ] Legal advocate/victim witness advocate
      [ ] Child therapy/support group
      [ ] Batterers' treatment program
      [ ] On-site social worker/advocate
      [ ] Religious leader/organization
      [ ] Battered women's program/shelter
      [ ] Battered women/s support group
      [ ] Alcohol/substance abuse counseling
      [ ] National DV/IPV Hotline
      [ ] Local DV/IPV hotline
      [ ] Police, sheriff, or other local law enforcement
      [ ] Housing, educational, job or financial assistance
      [ ] Other referral (describe): ____
      [ ] Other action (describe): ____

6. Is there a protocol for dealing with adult IPV at your clinic/practice? (check one)
   [ ] Yes, and widely used
   [ ] Yes, and used to some extent
   [ ] Yes, but not used
   [ ] No
   [ ] Unsure

7. Are you familiar with the policy of the Ministry of Health Malaysia regarding management of IPV victims?
   [ ] Yes
   [ ] No

8. Is a camera available at your workplace for photographing IPV victims' injuries?
   [ ] Yes - - Type:
      [ ] Polaroid or other instant camera,
      [ ] Digital,
      [ ] Other: _____
   [ ] No
   [ ] Unsure

9. Do you practice in a country where it is legally mandated to report IPV cases involving competent (non-vulnerable) adults?
   [ ] Yes
   [ ] No
   [ ] Unsure

10. For every IPV victim you have identified in the past 6 months, how often have you:

	Never	Seldom	Some-times	Nearly always	Always	N/A
a. Documented patient's statements re. IPV in chart	1	2	3	4	5	6
b. Used a body-map to document patient injuries	1	2	3	4	5	6
c. Photographed victim's injuries to include in chart	1	2	3	4	5	6
d. Notified appropriate authorities even though it is not mandated	1	2	3	4	5	6
e. Conducted a safety assessment for victim	1	2	3	4	5	6
f. Conducted a safety assessment for victim's children	1	2	3	4	5	6
g. Helped an IPV victim develop a safety plan	1	2	3	4	5	6
h. Contacted an IPV service provider	1	2	3	4	5	6
i. Offered validating or supportive statements	1	2	3	4	5	6
j. Provided basic information about IPV	1	2	3	4	5	6
k. Provided referral and/or resource information	1	2	3	4	5	6

11. Are IPV patient education or resource materials (posters, brochures, etc.) available at your practice site? (check one)

[ ] Yes, well displayed, and accessed by patients
[ ] Yes, well displayed, but not accessed by patients
[ ] Yes, but not well displayed
[ ] No
[ ] Unsure

12. Do you provide abused patients with IPV patient education or resource materials? (check one)

[ ] Yes, almost always
[ ] Yes, when it is safe for the patient
[ ] Yes, but only upon patient request
[ ] No, due to inadequate referral resources in the community
[ ] No, because I do not feel these materials are useful in general
[ ] No, other reason (specify) ____

13. Do you feel you have adequate adult IPV referral resources for patients **at your workplace** (including mental health referral)?

[ ] Yes
[ ] No
[ ] Unsure

14. Do you feel you have adequate knowledge of referral resources for patients **in the community** (including shelters or support groups) for adult IPV victims?

[ ] Yes
[ ] No
[ ] Unsure

Section F: Personal Experience

1. Have you ever experienced physical violence, sexual abuse, emotional abuse, intimidation, economic deprivation or threats of violence in an intimate partner relationship?

   [ ] Yes [ ] No

2. Have you ever witnessed physical violence, sexual abuse, or psychological abuse directed towards a family member?

   [ ] Yes [ ] No

Thank you for completing this survey.
