# Supplementary material for: Primary healthcare providers’ perceived preparedness to respond to intimate partner violence in the public primary healthcare setting: a cross-sectional study
Source: BMC Prim Care. 2025 Mar 31;26:92. doi: 10.1186/s12875-025-02793-2 (PMC11956219; doi:10.1186/s12875-025-02793-2)
Supplement: Supplementary file 3 — Supplementary Material 3 [file 12875_2025_2793_MOESM3_ESM.docx]

**Table S1** Distribution and mean scores for items of perceived preparedness to respond to IPV among PHCPs (n=1505)

| **Statement** | **Not prepared n (%)** | **Minimally prepared n (%)** | **Slightly prepared n (%)** | **Moderately prepared n (%)** | **Fairly well prepared n (%)** | **Well prepared n (%)** | **Quite well prepared n (%)** | **Mean** | **SD** |
| --- | --- | --- | --- | --- | --- | --- | --- | --- | --- |
| 1. Ask appropriate questions about IPV based on patient's background, presenting conditions and circumstances | 91 (6.0) | 127 (8.4) | 250 (16.6) | 392 (26.0) | 273 (18.1) | 278 (18.5) | 94 (6.2) | 4.22 | 1.58 |
| 2. Appropriately respond to disclosures of abuse | 59 (3.9) | 115 (7.6) | 219 (14.6) | 372 (24.7) | 310 (20.6) | 317 (21.1) | 113 (7.5) | 4.44 | 1.53 |
| 3. Identify IPV indicators based on patient history, and physical examination | 70 (4.7) | 132 (8.8) | 243 (16.1) | 369 (24.5) | 324 (21.5) | 276 (18.3) | 91 (6.0) | 4.29 | 1.53 |
| 4. Assess an IPV victim's readiness to change to solve the problem | 77 (5.1) | 127 (8.4) | 254 (16.9) | 385 (25.6) | 311 (20.7) | 267 (17.7) | 84 (5.6) | 4.24 | 1.53 |
| 5. Help an IPV victim assess his/her danger of lethality | 68 (4.5) | 124 (8.2) | 213 (14.2) | 372 (24.7) | 320 (21.3) | 287 (19.1) | 121 (8.0) | 4.39 | 1.56 |
| 6. Conduct a safety assessment for the victim's children | 57 (3.8) | 108 (7.2) | 201 (13.4) | 392 (26.0) | 313 (20.8) | 310 (20.6) | 124 (8.2) | 4.48 | 1.52 |
| 7. Help an IPV victim create a safety plan | 85 (5.6) | 129 (8.6) | 218 (14.5) | 371 (24.7) | 317 (21.1) | 293 (19.5) | 92 (6.1) | 4.30 | 1.57 |
| 8. Document IPV history and physical examination findings in patient's file | 74 (4.9) | 121 (8.0) | 220 (14.6) | 331 (22.0) | 344 (22.9) | 300 (19.9) | 115 (7.6) | 4.40 | 1.57 |
| 9. Make appropriate referrals for IPV | 54 (3.6) | 99 (6.6) | 193 (12.8) | 311 (20.7) | 333 (22.1) | 362 (24.1) | 153 (10.2) | 4.64 | 1.55 |
|  |  |  |  |  |  |  |  |  |  |

**Table S2** Distribution and mean scores for items of perceived knowledge of IPV among PHCPs (n=1505)

| **Statement** | **Nothing n (%)** | **Very little n (%)** | **A little n (%)** | **A moderate amount n (%)** | **A fair amount n (%)** | **Quite a bit n (%)** | **Very much n (%)** | **Mean** | **SD** |
| --- | --- | --- | --- | --- | --- | --- | --- | --- | --- |
| 1. Your legal reporting requirements for IPV | 63 (4.2) | 154 (10.2) | 217 (14.4) | 525 (34.9) | 263 (17.5) | 173 (11.5) | 110 (7.3) | 4.15 | 1.48 |
| 2. Signs or symptoms of IPV | 41 (2.7) | 116 (7.7) | 260 (17.3) | 504 (33.5) | 328 (21.8) | 184 (12.2) | 72 (4.8) | 4.20 | 1.35 |
| 3. How to document IPV in patient’s chart | 175 (11.6) | 190 (12.6) | 328 (21.8) | 469 (31.2) | 214 (14.2) | 90 (6.0) | 39 (2.6) | 3.52 | 1.47 |
| 4. Referral sources for IPV victims | 126 (8.4) | 194 (12.9) | 301 (20.0) | 474 (31.5) | 245 (16.3) | 119 (7.9) | 46 (3.1) | 3.70 | 1.46 |
| 5. Perpetrators of IPV | 95 (6.3) | 183 (12.2) | 294 (19.5) | 480 (31.9) | 285 (18.9) | 123 (8.2) | 45 (3.0) | 3.81 | 1.42 |
| 6. Relationship between IPV and pregnancy | 96 (6.4) | 168 (11.2) | 299 (19.9) | 480 (31.9) | 284 (18.9) | 132 (8.8) | 46 (3.1) | 3.84 | 1.42 |
| 7. Recognising the childhood effects of witnessing IPV | 119 (7.9) | 174 (11.6) | 272 (18.1) | 481 (32.0) | 255 (16.9) | 148 (9.8) | 56 (3.7) | 3.83 | 1.49 |
| 8. What questions to ask to identify IPV | 111 (7.4) | 204 (13.6) | 300 (19.9) | 479 (31.8) | 257 (17.1) | 109 (7.2) | 45 (3.0) | 3.71 | 1.43 |
| 9. Why a victim might not disclose IPV | 89 (5.9) | 203 (13.5) | 277 (18.4) | 484 (32.2) | 268 (17.8) | 137 (9.1) | 47 (3.1) | 3.82 | 1.43 |
| 10. Your role in detecting IPV | 80 (5.3) | 169 (11.2) | 288 (19.1) | 464 (30.8) | 301 (20.0) | 147 (9.8) | 56 (3.7) | 3.93 | 1.43 |
| 11. What to say and not say in IPV situations with a patient | 116 (7.7) | 182 (12.1) | 306 (20.3) | 498 (33.1) | 248 (16.5) | 116 (7.7) | 39 (2.6) | 3.72 | 1.42 |
| 12. Determining danger for a patient experiencing IPV | 70 (4.7) | 179 (11.9) | 280 (18.3) | 497 (33.0) | 292 (19.4) | 139 (9.2) | 48 (3.2) | 3.91 | 1.39 |
| 13. Developing a safety plan with an IPV victim | 130 (8.6) | 200 (13.3) | 305 (20.3) | 461 (30.6) | 249 (16.5) | 118 (7.8) | 42 (2.8) | 3.68 | 1.46 |
| 14. The stages an IPV victim experiences in understanding and changing his/her situation | 140 (9.3) | 200 (13.3) | 298 (19.8) | 475 (31.6) | 245 (16.3) | 109 (7.2) | 38 (2.5) | 3.64 | 1.46 |
|  |  |  |  |  |  |  |  |  |  |
